# Genomic variation in 3,010 diverse accessions of Asian cultivated rice

**DOI:** 10.1038/s41586-018-0063-9

**Published:** 2018-04-25

**Authors:** Wensheng Wang, Ramil Mauleon, Zhiqiang Hu, Dmytro Chebotarov, Shuaishuai Tai, Zhichao Wu, Min Li, Tianqing Zheng, Roven Rommel Fuentes, Fan Zhang, Locedie Mansueto, Dario Copetti, Millicent Sanciangco, Kevin Christian Palis, Jianlong Xu, Chen Sun, Binying Fu, Hongliang Zhang, Yongming Gao, Xiuqin Zhao, Fei Shen, Xiao Cui, Hong Yu, Zichao Li, Miaolin Chen, Jeffrey Detras, Yongli Zhou, Xinyuan Zhang, Yue Zhao, Dave Kudrna, Chunchao Wang, Rui Li, Ben Jia, Jinyuan Lu, Xianchang He, Zhaotong Dong, Jiabao Xu, Yanhong Li, Miao Wang, Jianxin Shi, Jing Li, Dabing Zhang, Seunghee Lee, Wushu Hu, Alexander Poliakov, Inna Dubchak, Victor Jun Ulat, Frances Nikki Borja, John Robert Mendoza, Jauhar Ali, Jing Li, Qiang Gao, Yongchao Niu, Zhen Yue, Ma. Elizabeth B. Naredo, Jayson Talag, Xueqiang Wang, Jinjie Li, Xiaodong Fang, Ye Yin, Jean-Christophe Glaszmann, Jianwei Zhang, Jiayang Li, Ruaraidh Sackville Hamilton, Rod A. Wing, Jue Ruan, Gengyun Zhang, Chaochun Wei, Nickolai Alexandrov, Kenneth L. McNally, Zhikang Li, Hei Leung

**Affiliations:** 1 Institute of Crop Sciences, Chinese Academy of Agricultural Sciences, Beijing, China; 2 International Rice Research Institute, Manila, Philippines; 3 School of Life Sciences and Biotechnology, Shanghai Jiao Tong University, Shanghai, China; 4 BGI Genomics, BGI-Shenzhen, Shenzhen, China; 5 Agricultural Genomics Institute, Chinese Academy of Agricultural Sciences, Shenzhen, China; 6 Shenzhen Institute for Innovative Breeding, Chinese Academy of Agricultural Sciences, Shenzhen, China; 7 Anhui Agricultural University, Hefei, China; 8 Arizona Genomics Institute, School of Plant Sciences, University of Arizona, Tucson, AZ USA; 9 China Agricultural University, Beijing, China; 10 Institute of Genetics and Developmental Biology, Chinese Academy of Sciences, Beijing, China; 11 DOE Joint Genome Institute, Walnut Creek, CA USA; 12 Lawrence Berkeley National Laboratory, Berkeley, CA USA; 13 Advanced Science and Technology Institute, Department of Science and Technology, Quezon City, Philippines; 14 UMR AGAP, CIRAD, Montpellier, France; 15 UMR AGAP, Université de Montpellier, Montpellier, France; 16 Shanghai Center for Bioinformation Technology, Shanghai, China

**Keywords:** Agricultural genetics, Natural variation in plants

## Abstract

Here we analyse genetic variation, population structure and diversity among 3,010 diverse Asian cultivated rice (*Oryza sativa* L.) genomes from the 3,000 Rice Genomes Project. Our results are consistent with the five major groups previously recognized, but also suggest several unreported subpopulations that correlate with geographic location. We identified 29 million single nucleotide polymorphisms, 2.4 million small indels and over 90,000 structural variations that contribute to within- and between-population variation. Using pan-genome analyses, we identified more than 10,000 novel full-length protein-coding genes and a high number of presence–absence variations. The complex patterns of introgression observed in domestication genes are consistent with multiple independent rice domestication events. The public availability of data from the 3,000 Rice Genomes Project provides a resource for rice genomics research and breeding.

## Abstract (web summary)

Analyses of genetic variation and population structure based on over 3,000 cultivated rice (*Oryza sativa*) genomes reveal subpopulations that correlate with geographic location and patterns of introgression consistent with multiple rice domestication events.

## Main

Asian cultivated rice is grown worldwide and comprises the staple food for half of the global population. It is envisaged that by the year 2035^1^ feeding this growing population will necessitate that an additional 112 million metric tons of rice be produced on a smaller area of land, using less water and under more fluctuating climatic conditions, which will require that future rice cultivars be higher yielding and resilient to multiple abiotic and biotic stresses. The foundation of the continued improvement of rice cultivars is the rich genetic diversity within domesticated populations and wild relatives^2–4^. For over 2,000 years, two major types of *O. sativa*—*O. sativa* Xian group (here referred to as *Xian*/Indica (XI) and also known as , *Hsien* or Indica) and *O. sativa* Geng Group (here referred to as *Geng*/Japonica (GJ) and also known as , *Keng* or Japonica)*—*have historically been recognized^5–7^. Varied degrees of post-reproductive barriers exist between XI and GJ rice accessions^8^; this differentiation between XI and GJ rice types and the presence of different varietal groups are well-documented at isozyme and DNA levels^6,9^. Two other distinct groups have also been recognized using molecular markers^10^; one of these encompasses the Aus, Boro and Rayada ecotypes from Bangladesh and India (which we term the circum-Aus group (cA)) and the other comprises the famous Basmati and Sadri aromatic varieties (which we term the circum-Basmati group (cB)).

Approximately 780,000 rice accessions are available in gene banks worldwide^11^. To enable the more efficient use of these accessions in future rice improvement, the Chinese Academy of Agricultural Sciences, BGI-Shenzhen and International Rice Research Institute sequenced over 3,000 rice genomes (3K-RG) as part of the 3,000 Rice Genomes Project^12^.

Here we present analyses of genetic variation in the 3K-RG that focus on important aspects of *O. sativa* diversity, single nucleotide polymorphisms (SNPs) and structural variation (deletions, duplications, inversions and translocations). We also construct a species pan-genome consisting of ‘core’ genes that are present in all individuals and ‘distributed’ (variable, accessory or dispensable) genes that are absent in some individuals^13,14^. The gene presence–absence variations (PAVs) represent another component of species genetic diversity. Our analyses provide new perspectives on rice intra-species diversity and evolutionary history.

## Genome mapping, size and SNP variation

Baseline genome sequencing, analyses, and accession information and metadata for the 3,024 rice genomes are summarized in Supplementary Data 1 and Supplementary Notes. Fourteen accessions were excluded from further analyses after quality control. The remaining 3,010 genomes had an average mapping coverage of 92% (74.6–98.7%) (Supplementary Data 2 Table 1), when aligned to the *O. sativa* cv. Nipponbare IRGSP 1.0 reference genome^15^ (hereafter referred to as ‘Nipponbare RefSeq’). The estimated size of the genome was 375.1 ± 20.9 Mb, with 42.5 ± 0.4% guanine–cytosine content and 35.6 ± 3.7% repetitive sequence content (Supplementary Data 3 Table 1).

**Extended Data Fig. 1:**
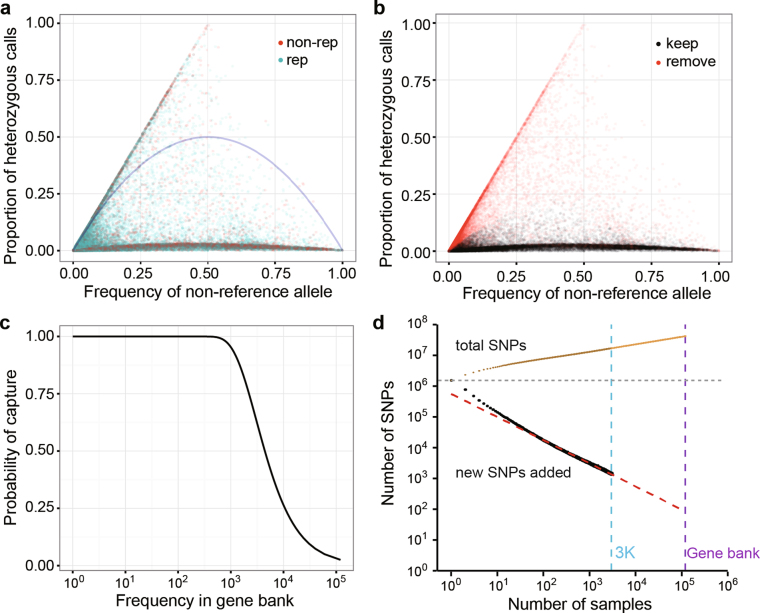
SNP filtering, discovery rate, and projected discovery upon further sequencing. **a**, Proportion of heterozygous calls versus allele frequency. Each dot represents a SNP from a random sample of 100,000 SNPs. Blue curve shows theoretical Hardy–Weinberg equilibrium. The points have opacity of 5%, such that regions with higher point densities are highlighted. The bulk of SNPs lie on the Hardy–Weinberg equilibrium curve scaled by a factor of about 0.05, which implies a Wright’s inbreeding coefficient of *F* = 0.95. **b**, The same plot with colour representing the outcome of filtering. The SNPs that are marked ‘keep’ (black) form the base SNP set. **c**, The estimated proportion of gene bank SNPs captured by 3K-RG samples, per frequency. The 3,010 samples capture more than 99.99% of gene-bank SNPs of frequency greater than 0.25%. **d**, Projected new SNP discovery rate based on simulations. For a given number of samples (*x* axis), the graph shows estimated mean number of new SNPs discovered in the last sample.

We identified over 29 million SNPs—27 million of which are bi-allelic—and found high concordance (>96%) with previous reports (Supplementary Notes)^16,17^. Filtering reduced this to a ‘base SNP’ set of approximately 17 million SNPs, which captured >99.9% of all SNPs with minor allele frequencies (MAF) > 0.25% (Extended Data Fig. 1). Half (56%) of non-transposable element (NTE) genes and the majority (91%) of transposable element (TE)-related genes have high-effect SNPs (Supplementary Data 2 Tables 2–4). NTE genes contained about 1.44 million moderate-to-high effect, and about 1.5 million low-effect, SNPs, which gave a ratio of 0.95 for moderate-to-high:low SNPs. For small indels, insertions affected 28% of NTE- and 50% of TE-related genes: deletions affected 41% of NTE- and 70% of TE-related genes. A typical genome in a major varietal group contains approximately 2 million (XI and cA), 0.3–0.8 million (GJ; depending on the subpopulation) or about 1.2 million (cB) SNPs (Supplementary Data 2 Table 5). The SNPs of a typical genome were classified as 7.9% moderate-to-high effect and 5.1% low effect.

## Population structure and diversity

**Fig. 1:**
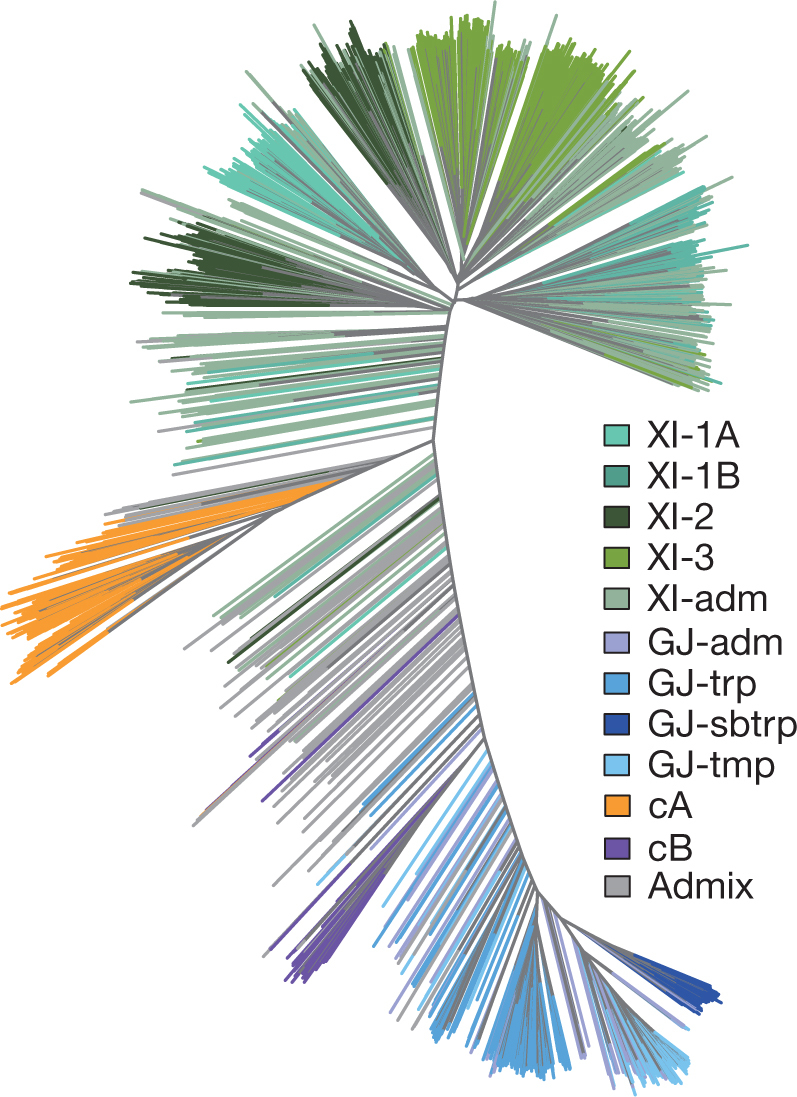
Unweighted neighbour-joining tree based on 3,010 samples and computed on a simple matching distance matrix for filtered SNPs. Samples are coloured by their assignment to *k* = 9 subpopulations from ADMIXTURE^46^.

**Extended Data Fig. 2:**
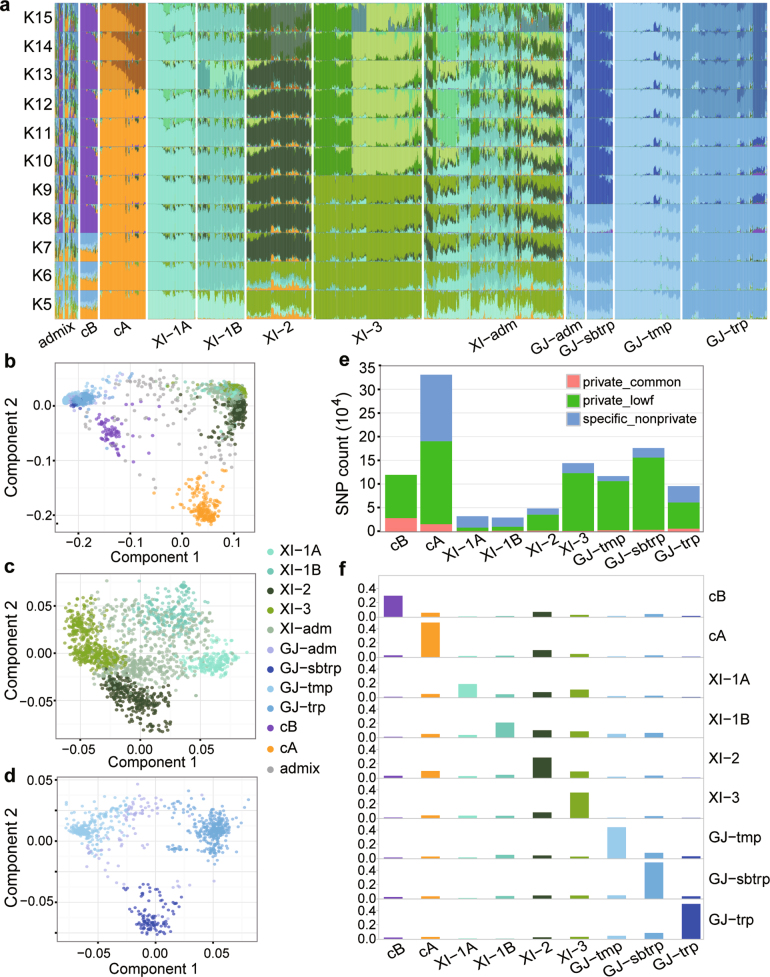
Population structure and subpopulation differentiation. **a**, ADMIXTURE analyses for *k* = 5 to *k* = 15. **b**–**d**, Multidimensional scaling plots for all (*n* = 3,010) (**b**), XI (*n* = 1,786) (**c**) and GJ (*n* = 849) (**d**) accessions. **e**, Private and specific SNPs in each subpopulation. Private alleles are defined as being present in at least 4 accessions in a subpopulation and not found in other subpopulations; population-specific alleles are common in the subpopulation (≥20%) but of low frequency (<2%) in others. **f**, Doubleton sharing—that is, SNPs shared by two accessions—within and between subpopulations, with values normalized by the sample sizes.

The 3K-RG accessions were classified into nine subpopulations (Fig. 1 and Extended Data Fig. 2a–d), most of which could be connected to geographic origins (Supplementary Data 1). There were four XI clusters (XI-1A from East Asia, XI-1B of modern varieties of diverse origins, XI-2 from South Asia and XI-3 from Southeast Asia); three GJ clusters (primarily East Asian temperate (named GJ-tmp), Southeast Asian subtropical (named GJ-sbtrp) and Southeast Asian tropical (named GJ-trp)); and single groups for the mostly South Asian cA and cB accessions. Accessions with admixture components <0.65 within XI and GJ were classified as ‘XI-adm’ and ‘GJ-adm’, respectively, and accessions that fell between major groups were classified as admixed (Extended Data Fig. 2a).

**Extended Data Fig. 3:**
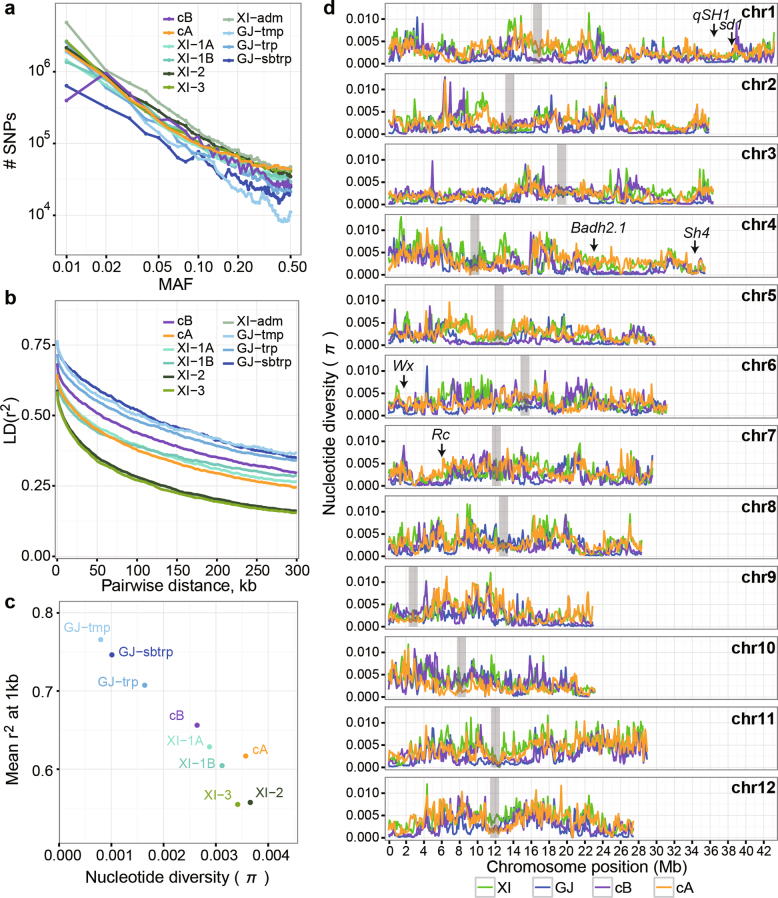
Genetic diversity within subpopulations. **a**, MAF histogram. **b**, Genome-wide linkage disequilibrium. **c**, Nucleotide diversity versus linkage disequilibrium. **d**, Diversity scans (*π*) for all chromosomes for major groups (XI, GJ, cA and cB) using 100-kb windows in which centromeric regions are highlighted in grey.

Distinct allele frequency profiles for SNPs of MAF > 10% occurred for the nine subpopulations with deviations from the neutral model reflecting different adaptations and demographic events (Extended Data Fig. 3a). Larger numbers of ‘private’ alleles were found in cA and cB than in other subpopulations (Extended Data Fig. 2e). Comparatively, XI subpopulations have smaller numbers of private alleles, probably owing to ongoing gene flow from natural hybridization and breeding. Doubleton sharing patterns within and between subpopulations showed the same trend (Extended Data Fig. 2f).

Linkage disequilibrium decay rates for combined subpopulations were higher in XI than GJ, with little variation between the two GJ subpopulations, as previously reported^7,16,18^. However, for the nine subpopulations, linkage disequilibrium decay between XI subpopulations varied more markedly, with XI-2 and XI-3 exhibiting faster linkage disequilibrium decay than XI-1A and XI-1B (Extended Data Fig. 3b). Furthermore, linkage disequilibrium decay correlates strongly with nucleotide diversity (*π*) among the nine subpopulations (*R*^2^ = 0.93, *P* value = 2.5 × 10^−5^) (Extended Data Fig. 3c).

**Fig. 2:**
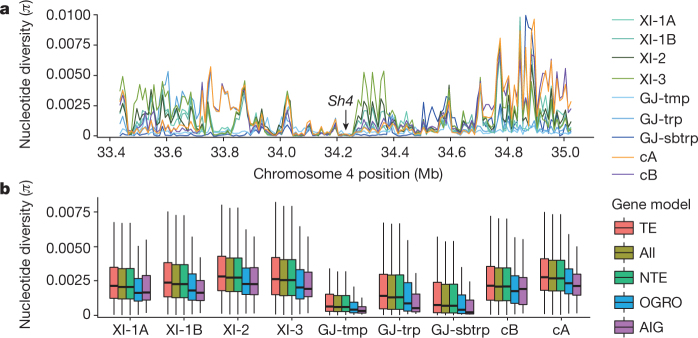
Nucleotide diversity. **a**, Differential nucleotide diversity between subpopulations at the *Sh4* locus on chromosome 4 using 10-kb sliding windows. **b**, Box plots of the distribution of *π* in 100-kb regions surrounding gene models across the genome. Box plots are shown for *k* = 9 subpopulations for all 100-kb windows (All) (*n* = 3,728 in total) and those containing genes annotated as transposable elements (TE) (*n* = 3,305 windows), NTE (*n* = 3,709), from the OGRO/QTARO database (OGRO) (*n* = 828) and the subset of 78 domestication-related genes (AIG) (*n* = 61 windows). Box plots show the median, box edges represent the first and third quartiles, and the whiskers extend to farthest data points within 1.5× interquartile range outside box edges.

Nucleotide diversity computation identified many regions of low genetic diversity that contained small numbers of genes under selective constraints (Extended Data Fig. 3d). *Sh4*^19^, which controls non-shattering, showed an accordant profile of diversity reduction across all subpopulations (Fig. 2a) that indicates much longer selection, when compared to *qSH1*^20^. At the semi-dwarf gene *sd1*^21^ locus, a narrow region of reduced diversity occurred in all major groups, which is a similar pattern to that observed for *qSH1*. However, higher diversity in the surrounding 100-kb regions occurred in the cA, cB and XI groups, whereas the GJ groups had extended regions of reduced diversity, which reflects the breeding history associated with the ‘green revolution’^22^. Different patterns of diversity reduction were observed at other important loci. The *Wx*^23^ locus that affects amylose content and stickiness on cooking, the *Badh2.1*^24^ locus that affects aroma and their surrounding regions are highly diverse in the XI, cA and cB groups, which indicates complex histories for selection for different types of eating quality; by contrast, both loci and their surrounding regions show low diversity in GJ. The *Rc*^25^ locus has very low diversity in all variety groups, with variable diversity in the surrounding regions in XI, cA and cB.

We compared SNP variation among TE-related genes, NTE-related genes, 1,021 genes with validated functions curated in the OGRO/QTARO database^26,27^ and a subset of 78 domestication and agronomically relevant genes (Supplementary Data 4). Genetic diversity was reduced significantly (*P* value < 10^−12^) near OGRO-curated genes and was often more extreme across the 78-gene subset in each subpopulation (Fig. 2b) when compared with all genomic regions containing genes, which suggests there may have been selection for these genes.

## Structural variations

**Fig. 3:**
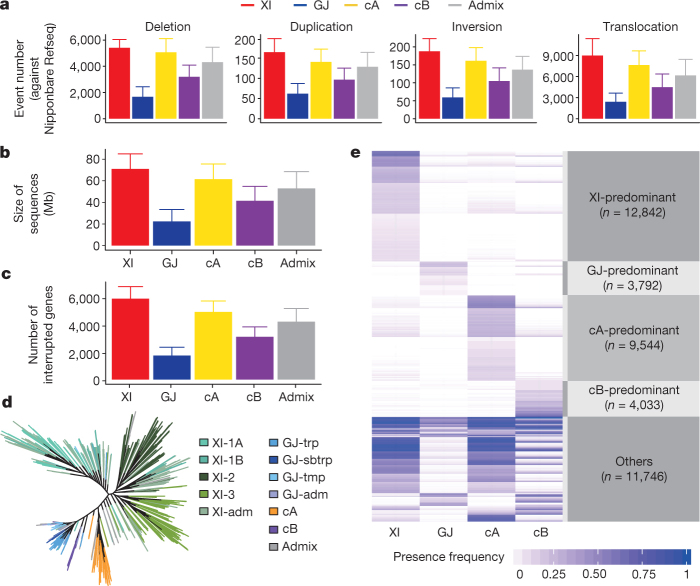
Summary of SVs for the 453 high-coverage rice accessions. **a**, Number of deletions, duplications, inversions and translocations. **b**, Genome sizes affected by SVs. **c**, Numbers of genes affected (included or interrupted) by the SVs. **d**, Phylogenetic relationship of 453 rice accessions built from 10,000 randomly selected SVs. **e**, Characterization of the 42,207 major-group-unbalanced SVs unevenly distributed among XI, GJ, cA and cB on the basis of two-sided Fisher’s exact tests. Bar plots in **a–c** are mean ± s.d. and numbers of accessions in XI, GJ, cA, cB and admix are 303, 92, 33, 10 and 15, respectively.

**Extended Data Fig. 4:**
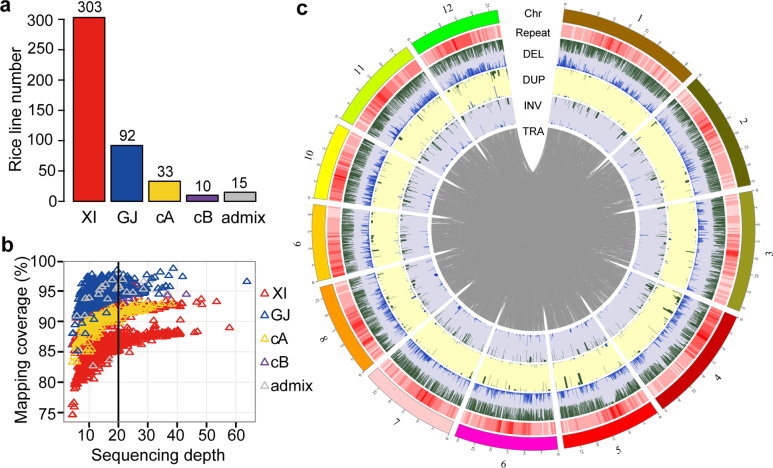
Selection of high-depth accessions and summary of SVs. **a**, Number of accessions with sequencing depths ≥ 20× and mapping depth ≥ 15×. **b**, Mapping coverage of the 3,010 rice genomes to the Nipponbare RefSeq as a function of sequence depth. **c**, Circular presentation of different types of structural variation detected in 453 high-coverage rice genomes when compared against the Nipponbare RefSeq. Chr, outermost circle represents 12 rice chromosomes with marks in Mb; Repeat, red heat map represents repeat content in 500-kb windows; DEL, green/blue colour with inner/outer bars represents the average frequencies of deletions detected in XI and GJ; DUP, green/blue colour with inner/outer bars represents the average frequencies of duplications detected in XI and GJ; INV, green/blue colour with inner/outer bars represents the average frequencies of inversions detected in XI and GJ; TRA, grey colour represents translocations across each genome with an average frequency > 0.3 in either XI or GJ.

Structural variations (SVs) were called for 3,010 accessions but we focused on 453 accessions with sequencing depths > 20× and mapping depths > 15×, because genome coverage stabilized when sequencing depths exceeded 20× (Extended Data Fig. 4a, b). We identified 93,683 SVs, including 582 SVs larger than 500 kb, with an average of 12,178 SVs per genome. The average sizes of the detected deletions, inversions and duplications are 5.3 ± 0.6 kb, 127.1 ± 19.4 kb and 105.1 ± 22.7 kb, respectively (Fig. 3a, Extended Data Fig. 4c and Supplementary Data 3 Table 2).

SVs showed very strong XI–GJ differentiation. On average, each XI accession differed from Nipponbare RefSeq by 14,754 SVs (8,990 translocations, 5,411 deletions, 188 inversions and 165 duplications), or 3.5× as many as in GJ accessions (Fig. 3a). On average, each cA or cB accession differed from Nipponbare RefSeq by 12,997 SVs and 7,892 SVs, respectively. The total SV sequence that differentiated two GJ accessions was about 22 Mb, whereas it reached 71 Mb between XI and GJ accessions (Fig. 3b). Notably, 1,940 SVs disrupted protein-coding genes within GJ, whereas >6,518 occurred between XI and GJ accessions (Fig. 3c). The SV phylogenetic tree (based on 453 accessions) is similar to the SNP tree, and clearly separates XI, GJ, cA and cB accessions (Fig. 3d). Moreover, the 41,957 major-group-unbalanced SVs that were distributed unevenly among XI, GJ, cA and cB accessions (Fig. 3e) accounted for 44.7% of all SVs and 41.0% of the 582 large SVs.

## Pan-genome and population differentiation

**Extended Data Fig. 5:**
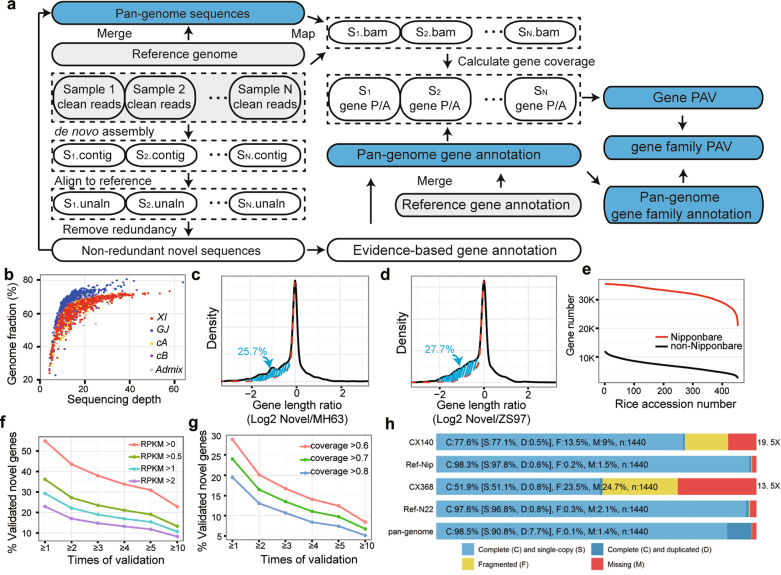
Map-to-pan strategy for rice pan-genome analyses. **a**, Map-to-pan pipeline for pan-genome analyses: (1) pan-genome sequences were derived by combining Nipponbare RefSeq and de novo assembled non-redundant novel sequences; (2) gene annotations were derived by combining Nipponbare RefSeq annotations and evidence-based gene predictions on novel sequences; (3) reads from each sample were mapped to pan-genome sequences; and (4) gene presence or absence was determined by coverage of mapped reads. Raw data for this pipeline is shown in grey boxes and the main output is shown in blue. P/A: presence/absence. **b**, Proportions of assembled genomes as a function of the sequencing depth (based on the Nipponbare RefSeq). **c**, **d**, Gene-length differences between the novel pan-genome genes and genes derived from the genome of Minghui 63 (MH63) (**c**) or Zhenshan 97 (ZS97) (**d**). Generally, the distribution should be symmetric: a ratio of > 0 means the novel gene is longer and a ratio < 0 means the novel gene is shorter. The dashed red lines show the symmetric distributions of the >0 part and the blue regions show the gene proportion with shorter lengths. **e**–**g**, Genomic (**e**) and transcriptomic (**f**, **g**) validation of novel genes. **e**, Validation based on genomic sequencing data, in which numbers of the Nipponbare RefSeq and non-Nipponbare RefSeq genes identified (>95% CDS coverage and >85% gene-body coverage) are shown against the numbers of supporting rice accessions in the 453 rice lines; **f**, **g**, Validation based on the mapping rates of the publicly available RNA sequencing data of rice, including gene expression (**f**) and coverage of the coding sequence (**g**). **h**, BUSCO evaluation for 1,440 highly conserved genes; CX140, the assembly of Illumina sequencing data of Nipponbare accessions; Ref-Nip, Nipponbare RefSeq. CX368, the assembly of Illumina sequencing data of accession N 22; Ref-N22, assembly of N 22 PacBio sequencing data.

The widespread SV and genome size variation (Supplementary Data 3 Tables 1 and 2) encouraged us to investigate the influence of PAVs on protein-coding genes across the 3K-RG. We first used a ‘map-to-pan’ strategy^28^ to build the species pan-genome (Extended Data Fig. 5a, b), by combining the Nipponbare RefSeq and non-redundant novel de novo assembled sequences; then, PAVs were determined by examining gene-body and coding sequence (CDS) coverage of mapped reads for each accession.

**Fig. 4:**
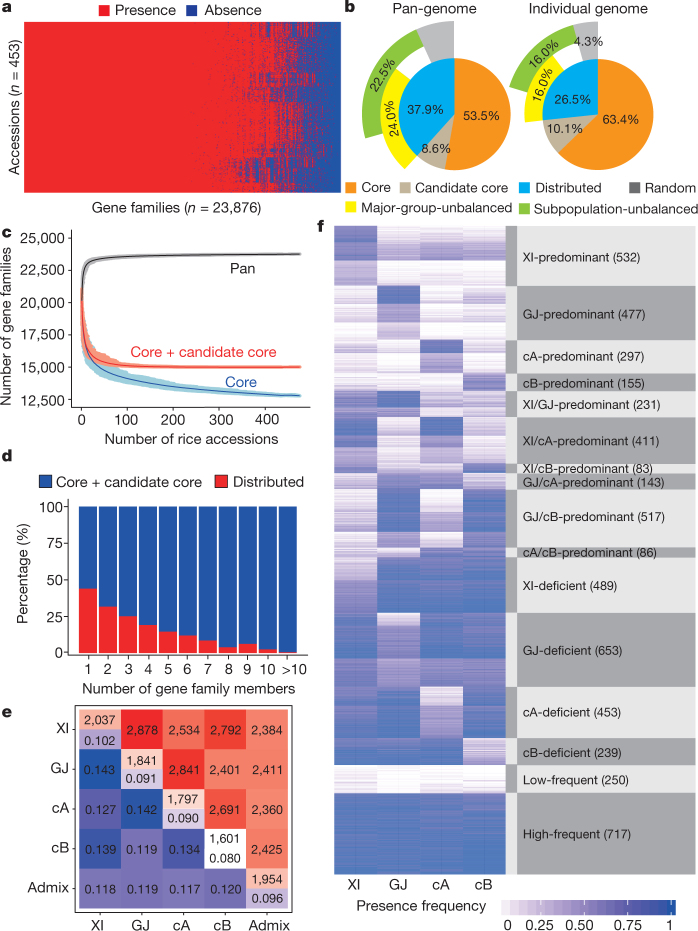
Pan-genome of *O. sativa*. **a**, Landscape of gene-family PAVs. Gene families were sorted by their occurrence and rice accessions were clustered with *k*-means method (*k* = 10). **b**, Compositions of the pan-genome and an individual genome. **c**, Simulation of the pan-genome and core genome based on 500 randomizations of rice genome orders. **d**, Proportions of the core and distributed gene families binned by gene family sizes. **e**, The average number of gene families that are different between two accessions. **f**, Characterization of 5,733 major-group-unbalanced gene families detected by two-sided Fisher’s exact tests.

**Extended Data Fig. 6:**
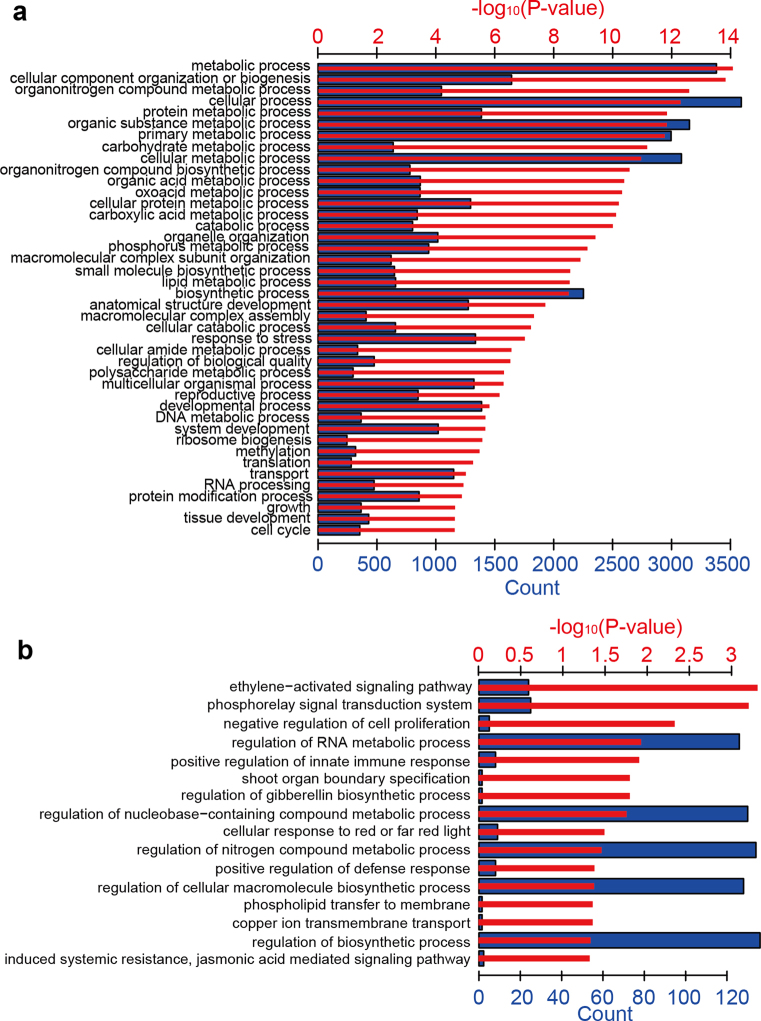
Representative enriched biological processes of core and distributed gene families. **a**, **b**, Representative enriched biological processes of core (**a**) and distributed gene families (**b**) are shown, with all terms sorted by their enriched *P* values (red bars). One-sided hypergeometric test built in the GOstats R package was used to calculate the *P* value of each GO term. The numbers of gene families involved in each GO term are shown in blue.

We identified a total 268-Mb non-redundant novel sequences of length >500 bp with <90% identity to Nipponbare RefSeq from assemblies of the 3,010 genomes, from which 12,465 novel full-length genes and several thousand novel genes with partial sequences were predicted. Nipponbare RefSeq genes and full-length novel genes could be merged into 23,876 gene families. The *O. sativa* core pan-genome was formed by 12,770 (53.5%) gene families present in all 453 high-coverage genomes, 2,056 (8.6%) without significant gene loss >1% (*P* value > 0.05) in all major groups formed candidate core gene families, and the remaining 9,050 (37.9%) comprised distributed gene families (Fig. 4a, b and Supplementary Data 3 Table 3). In silico simulation indicated these 9,050 gene families underestimate the distributed pan-genome (Fig. 4c). Hence, the *O. sativa* pan-genome consists of between 12,770 and approximately 14,826 (53.5% to about 62.1%) core gene families, and at least 9,050 (37.9%) distributed gene families: each accession contains between 63.4% and about 73.5% core gene families and at least 26.5% distributed gene families (Fig. 4b). The core gene families have more members (Fig. 4d) and represent essential gene families. Indeed, 5,476 (36.9%) core or candidate core gene families are enriched in essential functions for growth, development and reproduction (using Gene Ontology, GO), whereas only 862 (9.5%) of the distributed gene families could be annotated with GO terms, showing enrichment in regulation of immune and defence responses and ethylene metabolism (Extended Data Fig. 6a, b).

Pan-genome sequence coverage was evaluated using two new reference genomes^29^, IR 8 from the XI group and N 22 from the cA group (Supplementary Data 3 Table 4). We found 98.4% of the IR 8 and 98.6% of the N 22 genome sequences could be mapped to the pan-genome, whereas only 94.3% and 94.0% could be found in Nipponbare RefSeq. By comparing pan-genome data with high-quality XI reference genomes of Zhenshan 97 and Minghui 63^30^, approximately 25% of the novel genes were shorter owing to gene predictions from fragmented sequences (Extended Data Fig. 5c, d). Novel gene assemblies were validated by mapping raw reads of the 453 high-coverage genomes to the 12,465 novel genes; 11,792 genes (94.6%) had >95% CDS and >85% gene-body coverages were present in at least two rice lines. By comparison, 99.9% of Nipponbare RefSeq annotated genes were detected in the 453 high-coverage genomes (Extended Data Fig. 5e). Approximately 30% of the full-length novel genes were expressed with >1 read per kilobase per million reads in one or more of the 226 publicly available RNA sequencing datasets^31^ (Extended Data Fig. 5f, g). Further, benchmarking universal single-copy orthologues^32^ evaluation suggested little redundancy in predicted genes (Extended Data Fig. 5h).

**Extended Data Fig. 7:**
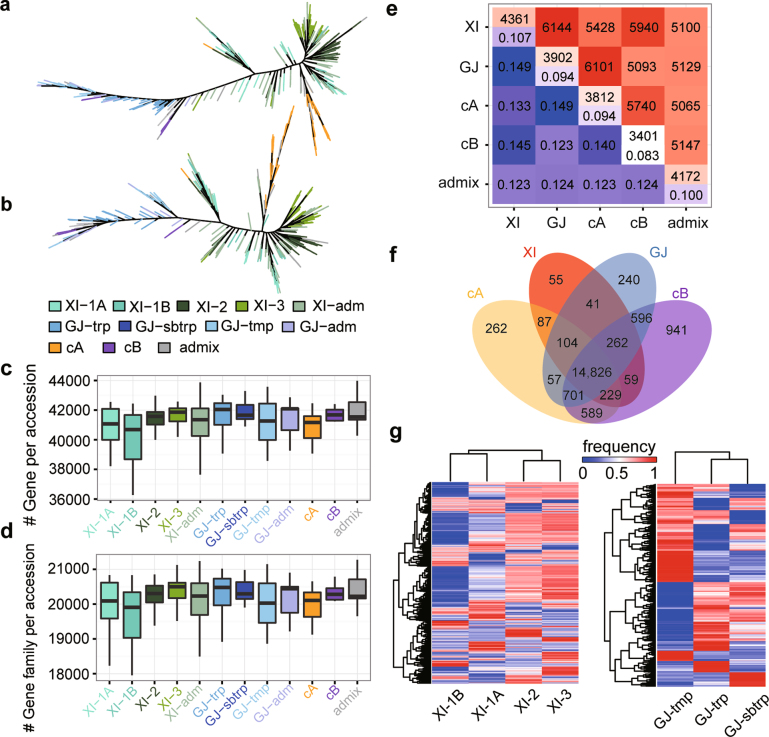
Characterization of gene or gene family presence or absence variations. **a**, **b**, Phylogenetic trees of the 453 rice accessions constructed on the basis of the presence or absence of the distributed genes (**a**) and gene families (**b**); both of which classified the 453 accessions into two major groups (XI and GJ), with each being further divided into several subpopulations that are tagged with different colours representing their classifications based on the SNP. **c**, **d**, Gene (**c**) or gene family (**d**) numbers per accession in different subpopulations; gene or gene family numbers were significantly different among XI subpopulations (Kruskal–Wallis tests, *P* value = 9.8 × 10^−8^ (gene) or 1.0 × 10^−6^ (gene family)). Box plots show the median, box edges represent the first and third quartiles and the whiskers extend to 1.5× interquartile range. **e**, The average number of genes that are different between two accessions in which all combinations of the 453 accessions were considered, and the proportions were calculated as the number of such differentiating genes adjusted by the gene numbers held in common by the two genome types. **f**, Venn diagram of the numbers of the core + candidate core gene families among the major groups of *O. sativa*. **g**, Cluster analysis of 4,270 XI-subpopulation-unbalanced gene families and 1,384 GJ-subpopulation-unbalanced gene families.

Analyses of the PAVs of genes (or gene families) were able to distinguish the major varietal groups, and show that there is considerable variation among and within subpopulations (Extended Data Fig. 7a–d). On average, major group accessions differ by about 4,000 (approximately 10%) genes and about 2,000 (approximately 10%) gene families, whereas XI and GJ accessions differ by more than 6,144 (about 14.9%) genes and 2,878 (14.3%) gene families (Fig. 4e and Extended Data Fig. 7e). The GJ pan-genome has 23,167 gene families comprising 46,115 genes, which makes it 1.9% smaller than XI in terms of gene families and 2.5% smaller in terms of genes. However, all GJ accessions have 240 core gene families (1,594 genes) in common, four times as many as in XI (Extended Data Fig. 7f). In addition, 5,733 major-group-unbalanced gene families were more frequent in some populations but lower in others, including hundreds of XI- and GJ-predominant gene families (Fig. 4f). Moreover, we identified 4,270 XI and 1,384 GJ subpopulation-unbalanced gene families, showing variation between subpopulations within each major group (Extended Data Fig. 7g).

## Evolution and domestication of rice

**Extended Data Fig. 8:**
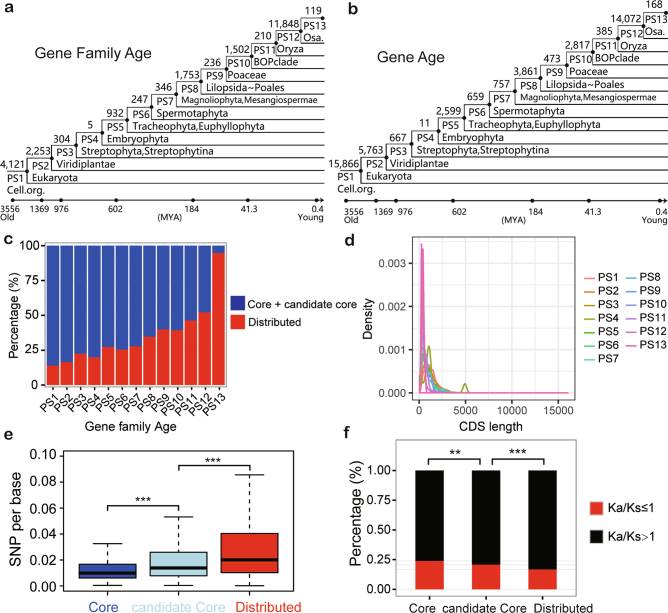
Evolution of the pan-genome of *O.sativa*. **a**, **b**, The numbers of gene families (**a**) and genes (**b**) that emerged at different evolutionary times, from PS1 (single-cell organisms) to PS13 (*O. sativa*). **c**, The age distribution of the core and distributed gene families. **d**, Coding sequence length distribution for Nipponbare RefSeq genes with different ages. **e**, **f**, SNP variation of the core and distributed genes against the Nipponbare RefSeq. **e**, The density of SNPs in the coding region of core and distributed genes in the 3,010 rice lines; SNP density in core genes is lower than that in candidate core genes (two-sided Wilcoxon test) and SNP density in candidate core genes is lower than that in distributed genes (two-sided Wilcoxon test). Box plots show the median, box edges represent the first and third quartiles, and the whiskers extend to 1.5× interquartile range. **f**, *K*_a_/*K*_s_ of the core and distributed genes. After removing genes with no synonymous SNPs, there are 3,144 core, 455 candidate core and 800 distributed genes with *K*_a_/*K*_s_ > 1 and 10,005 core, 1,727 candidate core and 3,957 distributed genes with *K*_a_/*K*_s_ < 1. Two sided *χ*-square tests were used to determine the difference of the proportions. **P < 0.01, ***P < 0.001.

To gain insights into the evolutionary history of the rice pan-genome, gene and gene family ages were estimated by aligning protein sequences to the NR protein database (ftp://ftp.ncbi.nlm.nih.gov/blast/db/) partitioned into 13 taxonomic levels (Extended Data Fig. 8a, b). We observed that: (1) new genes and gene families evolved at alternating rates from phylostratum 1 (PS1) (approximately 3.6 billion years ago) to the emergence of the terminal PS13 clade containing *O. sativa* (about 1.5 million years ago); (2) there was an explosive emergence of new genes accompanying the appearance of *Oryza* at PS12; (3) core genes tended to be more ancient, and most novel genes or gene families were younger and shorter (Extended Data Fig. 8c, d), consistent with recent reports for other species^33^; (4) significantly (*P* value < 0.001) higher SNP variation occurred in distributed genes than in core genes (0.0325 versus 0.0142 SNPs per base) (Extended Data Fig. 8e); and (5) a significantly (*P* value < 0.001) higher proportion of core genes were under negative selection as compared with those in the Nipponbare RefSeq (Extended Data Fig. 8f).

**Fig. 5:**
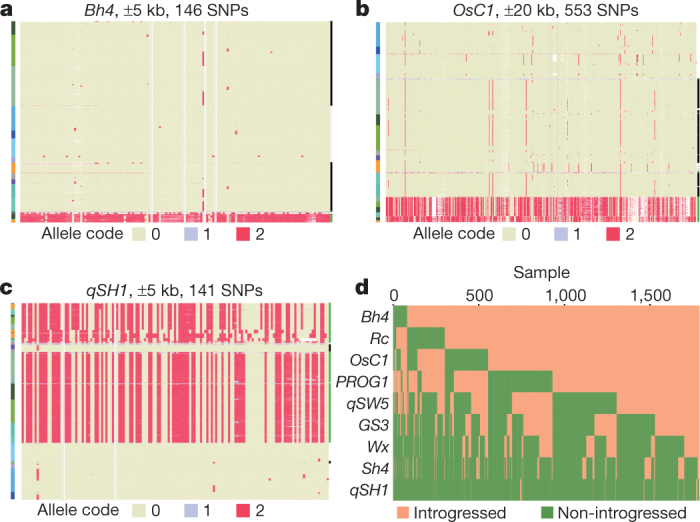
Haplotype analyses and introgression. **a–c**, Haplotypes around the domestication genes *Bh4*(**a**), *OsC1*(**b**) and *qSH1*(**c**). Rows correspond to samples and columns correspond to SNPs. Grey vertical lines mark the gene position. Left colour bar represents the *k* = 9 subpopulations. Right colour bar shows introgression status of the XI samples (green, no introgression; black, putative introgression from GJ). **d**, A heat map showing results of an introgression test of 1,789 XI samples at each of the nine domestication genes. *y* axis, genes; *x* axis, XI samples.

**Extended Data Fig. 9:**
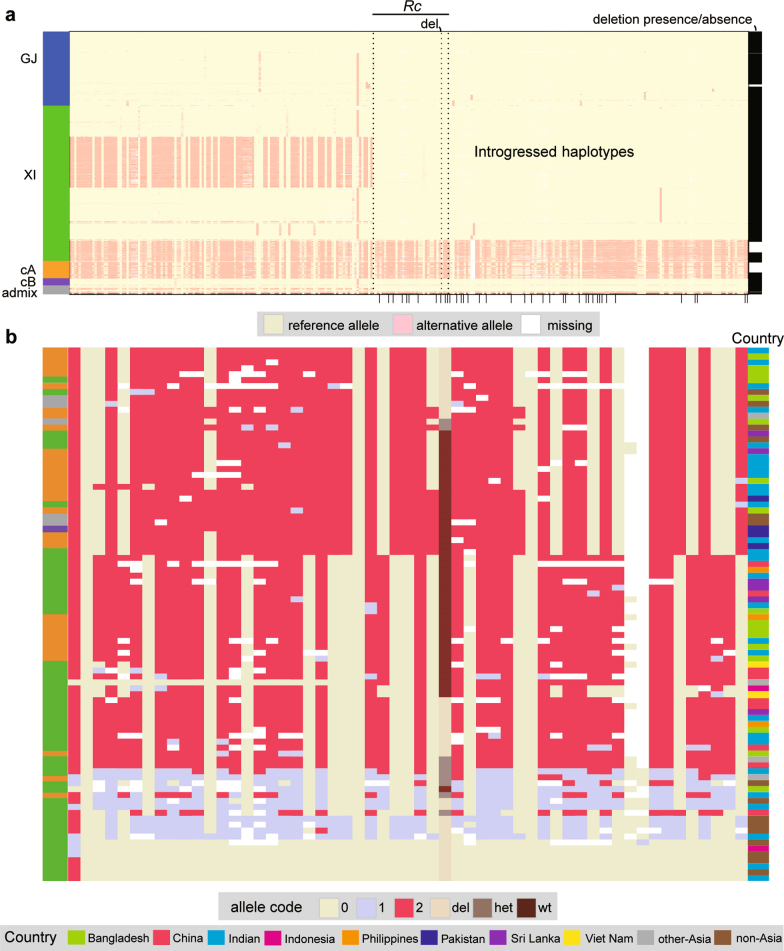
Haplotypes around the red pericarp (*Rc*) gene. Rows correspond to samples and columns correspond to SNPs. The colour of the rectangle denotes the number of non-Nipponbare alleles in a genotype. **a**, Haplotypes of 3,010 samples in a region ±25 kb around *Rc* show the presence of many distinctly non-GJ haplotypes that carry either the wild-type or domesticated allele at the causative deletion site. **b**, Zoomed-in view of a subset of 90 samples and 53 SNPs within the *Rc* gene and 15-kb downstream highlights the wide dispersal of non-GJ domesticated haplotypes.

Regarding *O. sativa* domestication, we constructed haplotype plots for nine important domestication genes—*Rc*^25^, *Bh4*^34^, *PROG1*^35^, *OsC1*^36^, *Sh4*^19^, *Wx*^23^, *GS3*^37^, *qSH1*^20^ and *qSW5*^38^ (Fig. 5a–c and Extended Data Fig. 9). Although a large number of XI samples carry an allele found in GJ, many XI accessions carry alleles at each of these loci that are absent in GJ (Fig. 5d). In fact, about 70% of XI accessions do not carry GJ introgressions in at least four genes, and only one XI sample (out of 1,789) had introgressed GJ haplotypes at all nine genes. This observation supports a model of independent domestication of some of the XI pool, rather than the simpler GJ-to-XI introgression hypothesis^2^. Furthermore, the 14-bp deletion in *Rc*^25^ for domesticated white pericarp was found in several XI lines that carried non-introgressed haplotypes (Extended Data Fig. 9), which suggests independent selection in part of the XI gene pool before introgression of the GJ haplotype became widespread in XI.

## Utility of the 3K-RG panel

**Extended Data Fig. 10:**
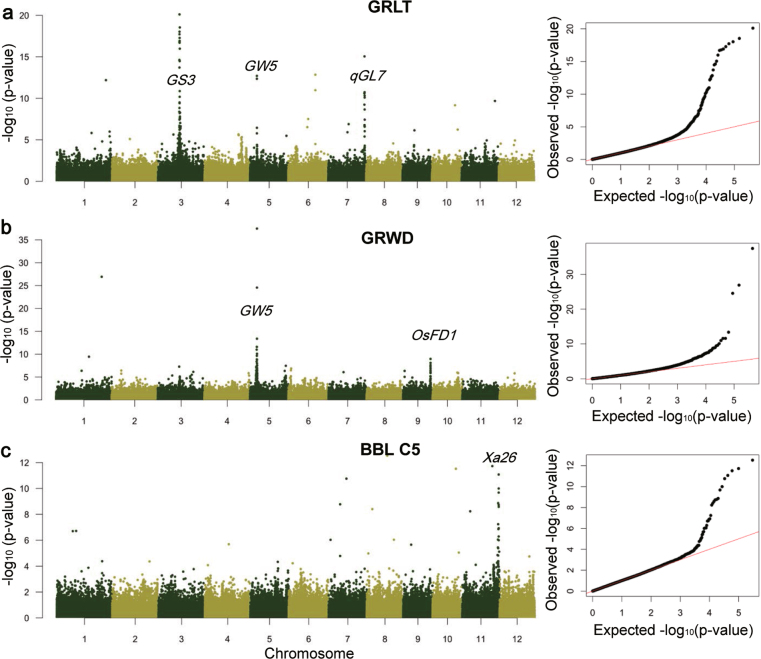
Genome-wide association for grain length, grain width and bacterial blight isolate C5. **a**–**c**, GWAS for grain length (GRLT, *n* = 2,012) (**a**), grain width (GRWD, *n* = 2,012) (**b**) and bacterial blight isolate C5 (BBL C5, *n* = 381) (**c**). GWAS was performed using filtered and linkage disequilibrium-pruned SNPs for historical trait data on source accessions for grain length and grain width (223,743 SNPs) and for newly acquired lesion length data for bacterial blight isolate C5 (148,999 SNPs). Manhattan plots for linkage disequilibrium-pruned datasets are shown to the left and quartile–quartile plots for expected versus observed −log(*P*) values to the right. Major peaks are annotated for known gene loci.

**Extended Data Fig. 11:**
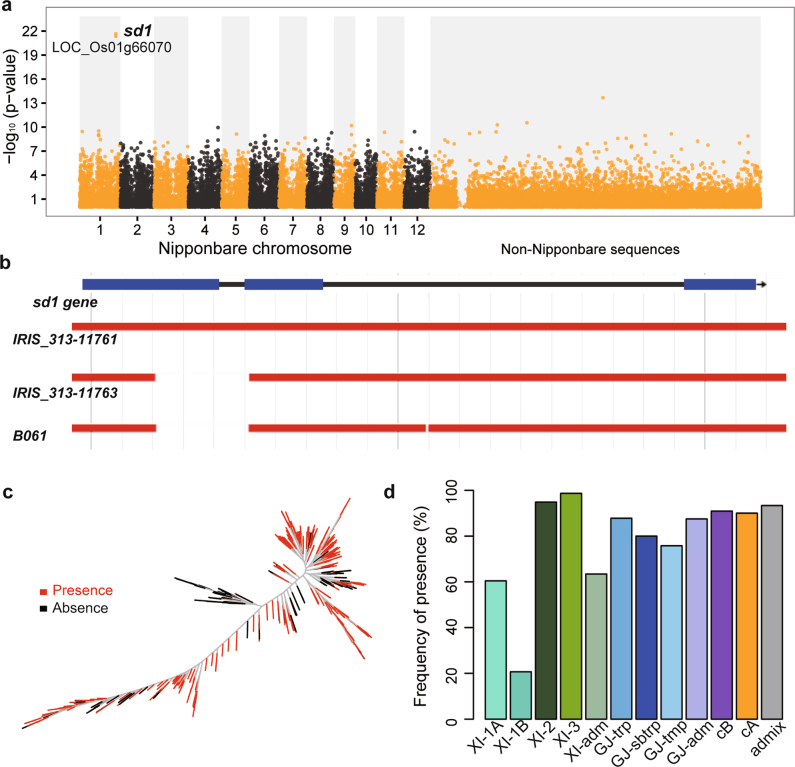
*sd1* gene and its correlation with plant height. **a**, The plot shows the correlation of the gene presence or absence variation with plant height (*n* = 323). The *P* values were calculated with Spearman’s correlation. **b**, Examples show that semi-dwarfism results from an approximately 385-bp deletion in the *sd1* locus. **c**, Distribution of the presence or absence of the *sd1* gene in the 453 rice accessions. **d**, *sd1* frequencies in rice subpopulations.

We demonstrated the use of the 3K-RG genomes and SNPs for trait mapping analyses for the highly heritable traits of grain length, grain width and bacterial blight resistance (Supplementary Notes). Major peaks for grain length with significantly (*P* value < 10^−10^) associated markers are on chromosomes 1, 3, 5, 6 and 7, and minor peaks are on chromosomes 4, 9, 10 and 11 (Extended Data Fig. 10a). Major peaks for grain width are found on chromosomes 1 and 5, with minor peaks on chromosomes 3 and 9 (Extended Data Fig. 10b). Genome-wide association study (GWAS) peaks were concordant with known loci, including *GS3*^37^, *GW5*^39^, and *qGL7*^40^ for grain length, and *GW5* for grain width. For grain width, the chromosome 9 novel peak coincides with *OsFD1*^41^, which codes for a bZIP transcription factor involved in flowering time and developmental plasticity (its pleiotropic regulatory function may therefore also affect grain width). Twelve peaks were detected for bacterial blight resistance to strain C5 of *Xanthomonas oryzae*, with the largest clustered around the resistance gene *Xa26*^42^ on chromosome 11 (Extended Data Fig. 10c). Moreover, correlation between gene PAVs and plant height detected the well-known green revolution gene (*sd1*) as the first-ranked candidate. *sd1* is classified as a distributed gene—caused by an approximately 385-bp deletion—and is significantly (*P* value < 10^−20^) associated with greatly reduced plant height; it was absent most frequently in XI-1A and XI-1B varieties (Extended Data Fig. 11).

## Discussion

We characterized genetic variation in the 3,010 sequenced accessions of *O. sativa* and found a high level of genetic diversity in rice. Although the 3K-RG analysis is expected to identify nearly all polymorphisms with MAF > 1%, our simulations suggest that it includes <40% of rare bi-allelic SNPs (MAF < 1%) in the International Rice Gene bank at the International Rice Research Institute (Extended Data Fig. 1c). We also characterized structural variation, and found that the average number of SVs between pairs of XI genomes (>12,000) was similar to that between two high-quality reference XI genomes^30^. The vast majority were deletions and translocations distributed across the genome (Extended Data Fig. 4c). Medium-sized SVs (≥500 kb) were mostly inversions and duplications, and a large percentage of them (37.9%) occur differentially between XI and GJ. We speculate that large numbers of SVs may contribute to the varying degrees of hybrid sterility and hybrid breakdown between XI and GJ accessions^43^. We also report pan-genome analyses for *O. sativa*, and the high numbers of PAVs highlight another component of within-species diversity for rice.

Our analysis brings more resolution to the within-species diversity of *O. sativa* (Extended Data Fig. 8e). Larger pan-genomes occur in XI than GJ accessions, but GJ accessions have more core genes than XI (Supplementary Data 3 Table 3), a result that was expected given the greater diversity within XI than GJ. This may relate to differences in eco-geographical distribution: GJ accessions experience harsher high- altitude and/or high-latitude environments, versus the less harsh but more diverse environments experienced by XI rice. Understanding the major group/subpopulation-core, -unbalanced and -predominant gene functions is expected to shed light on environmental adaptation of rice variety groups over thousands of years.

Although the 3K-RG population structure analyses based on SNPs and SVs were consistent with the five major groups that were previously known, additional subpopulations in the XI and GJ groups were identified and were suggestive of nine subpopulations that are correlated with geographic origin. Large numbers of SNPs, genes and gene families, and SVs were found to be unique to or predominant in single subpopulations. Varying patterns of diversity reduction across different rice subpopulations were observed in and around about 1,000 well-characterized genes. A closer look at patterns of haplotype sharing at domestication genes suggests that not all ‘domestication’ alleles came to XI from GJ. Taken together, our results—combined with archaeological evidence of XI cultivation for >9,000 years in both India and China^44,45^—support multiple independent domestications of *O. sativa*.

Our 3K-RG analysis highlights the genetic diversity that exists in rice germplasm repositories, and the usefulness of establishing a digital gene bank in which all accessions can be sequenced and catalogued. For example, we estimate that sequencing the rest of the gene bank of the International Rice Research Institute may enable the identification of >27 million additional SNPs (Extended Data Fig. 1d). The next challenge will be to examine associations of the 3K-RG genetic variation with agriculturally relevant phenotypes measured under multiple field and laboratory environmental conditions; this will guide and accelerate rice breeding by identifying genetic variation that will be useful in breeding efforts and future sustainable agriculture.

## Methods

No statistical methods were used to predetermine sample size. The experiments were not randomized and investigators were not blinded to allocation during experiments and outcome assessments.

### Sequencing data of the 3,000 Rice Genome project

The selection and sequencing of rice accessions have previously been described^12^. The SNPs/indels and SVs in 3,010 accessions were identified by mapping against the Nipponbare RefSeq, and the pan-genome sequence was created by integrating the Nipponbare RefSeq and non-redundant novel sequences derived from 3,010 rice assemblies. SV comparison and gene PAV analyses focused on 453 rice accessions with sequencing depth >20× and mapping depth >15× (Extended Data Figs. 4a, 5b).

### Detection of SNPs and indels

Reads were aligned to the Nipponbare RefSeq using BWA-MEM (release 0.7.10)^47^. The mapped reads were then sorted and duplicates were removed by Picard tools (release 1.119) (http://broadinstitute.github.io/picard/). The reads around indels were realigned by GATK RealignerTargetCreator and IndelRealigner package (release 3.2-2)^48^. The variants were called for each accession by the GATK UnifiedGenotyper (release 3.2-2)^48^ with ‘EMIT-ALL-SITES’ option. A joint genotyping step for comprehensive SNP union and filtering step was performed on the 3,010 emit-all-sites VCF files. A variant position is reported if at least one sample supports it with QUAL no less than 30. A total of 29,399,875 SNPs (27,024,796 are bi-allelic) and 2,467,043 indels (small insertions and deletions <40 bp) were identified from the analyses of the genomes of 3,010 accessions. Three subsets of the 3K-RG Nipponbare SNPs were defined using the following filtering criteria: (1) a base SNP set of ~17 million SNPs created from the ~27 million high-quality bi-allelic SNPs by removing SNPs in which heterozygosity exceeds Hardy–Weinberg expectation for a partially inbred species, with inbreeding coefficient estimated as 1−*H*_obs_/*H*_exp_, in which *H*_obs_ and *H*_exp_ are the observed and expected heterozygosity, respectively (detailed in Supplementary Notes); (2) a filtered SNP set of ~4.8 million SNPs created from the ~17-million-SNP base SNP set by removing SNPs with >20% missing calls and MAF < 1%; and (3) a core SNP set of SNPs derived from the filtered SNP set using a two-step linkage disequilibrium pruning procedure with PLINK^49,50^, in which SNPs were removed by linkage disequilibrium pruning with a window size of 10 kb, window step of one SNP and *r*^2^ threshold of 0.8, followed by another round of linkage disequilibrium pruning with a window size of 50 SNPs, window step of one SNP and *r*^2^ threshold of 0.8.

### Determining the effects of SNPs

The effects of all bi-allelic SNPs (low, medium and high effects) on the genome were determined based on the pre-built release 7.0 annotation from the Rice Genome Annotation Project (http://rice.plantbiology.msu.edu/) using SnpEff^51^ release 4.1l, with parameters -v -noLog -canon rice7. Using sequence ontology terms, a low-effect SNP was classified as ‘synonymous_variant’, ‘splice_region_variant’, ‘initiator_codon_variant’, ‘5_prime_UTR_premature_start_codon_gain_variant’ or ‘stop_retained_variant’. A moderate-effect SNP was identified as a ‘missense_variant’ and a high-effect SNP as a ‘start_lost’, ‘stop_gained’, ‘stop_lost’, ‘splice_donor_variant’ or ‘splice_acceptor_variant’. For indel effects, only indels with lengths that were not multiples of three were counted and SNPs overlapped with protein-coding regions (CDSs of RGAP 7^15^ genes) were considered as the most disruptive effects on genes. Results of the SNP and indel effect analysis are given in Supplementary Data 2 Tables 3, 4. We computed the SNP numbers (proportions) of rare SNPs and homozygous singletons for a ‘typical genome’ of a subpopulation as the median SNP number (proportion) of the SNPs in a given category among those genomes for that subpopulation (Supplementary Data 2 Table 5).

### Population structure and SNP diversity

Multi-dimensional scaling analysis was performed using the ‘cmdscale’ function in R, using the IBS distance matrix of the 3K-RG genomes computed with PLINK^49,50^ on the filtered SNP set. The same distance matrix was used to construct a phylogenetic tree by the unweighted neighbour-joining method, implemented in the R package phangorn^52^. The population structure of the 3K-RG dataset was analysed using ADMIXTURE software^46^ on the core SNP set (version 0.4, http://snp-seek.irri.org/download.zul). First, ADMIXTURE was run on 30 random 100,000-SNP subsets of the core SNP set with *k* (the number of groups) ranging from 5 to 18, and *k* = 9 was chosen because it was the minimal value of *k* to separate all previously known groups (cA, cB, XI, GJ-trp, GJ-tmp and part of GJ-sbtrp). With *k* = 9, ADMIXTURE was then run again on the whole core SNP set nine times with varying random seeds; the *Q*-matrices were aligned using CLUMPP software^53^ and clustered on the basis of similarity. Then, the matrices belonging to the largest cluster were averaged to produce the final matrix of admixture proportions. Finally, the group membership for each sample was defined by applying the threshold of ≥ 0.65 to this matrix. Samples with admixture components <0.65 were classified as follows. If the sum of components for subpopulations within the major groups XI and GJ was ≥ 0.65, the samples were classified as XI-adm or GJ-adm, respectively, and the remaining samples were deemed ‘fully’ admixed (admix). Branches of the phylogenetic tree were coloured according to the *k* = 9 admixture classification (Fig. 1).

We computed linkage disequilibrium decay in each subpopulation as follows. The value of *r*^2^ was computed for each pair of SNPs of frequency ≥ 10% in the respective subpopulations that are separated by at most 300 kb using PLINK. The distances were binned into 1-kb bins (separately for each chromosome) and the median value of *r*^2^ in each bin was taken. The medians for each chromosome were then averaged to produce a final *r*^2^ estimate for the bin. We computed nucleotide diversity (*π*) for non-overlapping 10-kb and 100-kb windows along the Nipponbare RefSeq by adopting an approach similar to VariScan^54^ for genome-wide DNA polymorphism analyses and implemented as a custom R script.

### Detection of genomic SVs and population differentiation

Genomic-SV detection for each of the 3,010 rice accessions was performed using a customized version of novoBreak^55^ (https://sourceforge.net/projects/novobreak/?source=navbar) against the Nipponbare RefSeq. SVs inferred by no less than 3 reads were further filtered with the following conditions: (1) more than four supporting split reads or (2) no fewer than three discordant read pairs. We detected deletions, inversions and duplications with sizes between 100 bp and 1 Mb, and translocations. Here, translocations were SVs with ‘inter-chromosomal breakpoints’. All SVs that passed the filter criteria in the 3K-RG accessions were pooled together. Two adjacent SVs were identified as the same SV if their start and end positions varied no more than 1 kb, and the overlapping region was more than 50% of the total size. The presence–absence matrix of SVs in each accession was built based on this pooled SV dataset. To obtain reliable SV comparison analysis results, we focused only on the 453 high-depth accessions (Extended Data Fig. 4a). Major-group-unbalanced SVs were determined by two-sided Fisher’s exact test followed by Benjamini–Hochberg adjustment (false discovery rate (FDR) < 0.05), similar to the detection of major-group-unbalanced genes.

### De novo assembly

A variation of SOAPdenovo2^56^ (version r240) with customized *k*-mers was used to assemble the rice genomes. A *k*-mer value was initially set for each accession according to a linear model ‘K=2*int (0.38*(sequencing depth) +10)+1’, which was trained from 50 randomly selected rice accessions. The best *k*-mer value was decided by checking the N50 of the SOAPdenovo results. The command line for SOAPdenovo was ‘SOAPdenovo-63mer (or SOAPdenovo-127mer) all -s configure_file (average insertion length set as 460 in the configure file) -K *k*-mer -R -F’ with iteration over different *k*-mers until N50 of the assembly with that *k*-mer is higher than those with ‘*k*-mer +2’ and ‘*k*-mer −2’. On average, we needed to run SOAPdenovo ~3.94 times for each rice accession. The quality of the genome assembly was evaluated for these contigs using QUAST version 2.3^57^.

### Sequencing and de novo assembly of IR 8 and N 22 reference genomes

High molecular weight DNA was extracted from young leaves adopting the protocol^58^ with minor modifications. The PacBio library was prepared following the 20-kb protocol (see ‘User-Bulletin-Guidelines-for-Preparing-20-kb-SMRTbell-Templates document.pdf’, available from https://www.pacb.com/support/documentation/?fwp_documentation_search="PN%20100-286-700-04") and was sequenced on an RSII sequencer with movie collection time of 6 h. The raw data of N 22 and IR 8 were assembled with FALCON^59^ and Canu^60^, respectively. Contigs were polished twice with PacBio raw reads using Quiver (https://github.com/PacificBiosciences/GenomicConsensus) and the IR 8 assembly was further polished with 66× WGS 2× 150-bp Illumina data using Pilon^61^. Polished contigs were assigned to pseudomolecules using Genome Puzzle Master^62^. Assembly statistics can be found in Supplementary Data 3 Table 4. IR 8 and N 22 were applied to evaluate the completeness and redundancy of the pan-genome.

### Pan-genome construction

SOAPdenovo assembly for each accession was assessed by QUAST^57^ with Nipponbare RefSeq as the reference. From QUAST output, unaligned contigs longer than 500 bp were retrieved and merged. CD-HIT version 4.6.1^63^ was used to remove redundant sequences at a cutoff of 90% identity with the command ‘-c 0.9 -T 16 -M 50000’. For remaining sequences, all-versus-all alignments with BLASTN were carried out to ensure that these sequences had no redundancy. Next, various contaminants including Archaea, bacteria, viruses, fungi and metazoans were removed. The non-redundant sequences were aligned to the NT database (downloaded from NCBI, 26 July 2014) with BLASTN with parameters ‘-evalue 1e-5 -best_hit_overhang 0.25 -perc_identity 0.5 -max_target_seqs 10’. Contigs of which the best alignments (considering *E*-values and identities) were not from Viridiplantae were considered as contaminants and were filtered out. The remaining contigs formed the non-redundant novel sequences. The rice species pan-genome was then generated by combining the Nipponbare RefSeq and non-redundant novel sequences.

### Annotation of the pan-genome

The gene–transcript annotation of the Nipponbare RefSeq was downloaded from the Rice Annotation Project^64^, and if a protein-coding gene contained multiple transcripts only the transcript with the longest open reading frame was selected as the representative for the gene. Protein-coding genes on novel sequences were predicted using MAKER^65^, a gene prediction tool combining ab initio predictions, expression evidence and protein homologies. In detail, repeats were first masked (soft mask for low-complexity repeats) with RepeatMasker (www.repeatmasker.org) and RepeatRunner^66^. Two ab initio predictors, SNAP^67^ and AUGUSTUS^68^, were called by MAKER^65^ to predict gene models with their default parameters for rice. All rice expressed sequence tags (ESTs) were downloaded from GenBank (15 December 2014) and were aligned to the novel sequences with BLASTN. All rice proteins were downloaded from NCBI (15 December 2014) and were aligned to the novel sequences with BLASTX. To obtain more informative alignments, Exonerate^69^ was used to realign each sequence identified by BLAST around splice sites. EVidenceModeller^70^ was used to combine and refine the ab initio predictions with RNA and protein evidence. Incomplete gene models were removed before the consequent analysis.

### Adjustment of predicted genes

We aligned the predicted transcripts against Nipponbare RefSeq to remove potential redundancy. Redundant genes were removed when the genes were clustered into gene families. However, when attempting to identify the number of novel genes, the redundant ones were removed first. We clustered all genes at a global identity of 95%, and removed novel genes that were not representative of the group.

### Evaluation of pan-genome redundancy

We ran BUSCO (benchmarking universal single-copy orthologues) v.2.0^32^ on CX140 (a Nipponbare accession) assembly, Nipponbare RefSeq, CX368 (an N 22 accession) assembly, N 22 high-quality reference genome and the pan-genome sequences. Augustus-3.2.3^68^ and hmmer-3.1b^71^ were used for gene prediction in BUSCO. BUSCO was run with genome mode with embryophyta_odb9 as a reference.

### Functional analysis

All protein sequences of pan-genome were extracted and aligned to the GO sequence database (http://geneontology.org/ on 4 April 2015) with BLASTP. Only alignments with *E*-values < 1 × 10^−5^ and identity > 0.3 were used. GO terms for each gene were estimated to be the same as those of its best-hit protein. In total, 20,842 (43.3%) genes could be annotated. For a gene family, its GO terms are the non-redundant set of the GO terms of the genes within this gene family. Overall, 6,338 (26.5%) gene families could be annotated. Enrichment of GO terms was carried out using the GOstats^72^ package in R with all gene families as the background.

### Validation of the non-Nipponbare RefSeq genes

We verified the novel genes by multiple approaches. First, for each gene, we examined the number of accessions that possessed it. We mapped the sequencing reads to the pan-genome sequences. Genes with CDS coverage over 0.95 and gene-body coverage over 0.85 were considered to be present. Second, we verified the novel genes with 226 RNA sequencing experiments from 17 projects^30^. RNA sequencing reads were first trimmed with Trimmomatic version 0.32^73^ with parameters ‘ILLUMINACLIP:2:30:10 LEADING:20 TRAILING:20 SLIDINGWINDOW:4:20 MINLEN:36’ and then aligned to the pan-genome sequences with a split-aligner HISAT2 version 2.0.1-beta^74^ using default parameters. The coverage of each gene was calculated with ‘BEDtools coverage’ in BEDtools suite version 2.17.0^75^.

### Gene family annotation

The genes were clustered to gene families with OrthoMCL version 2.0.9^76^. All genes were extracted and translated into protein sequences and the protein sequences were compared by using all-by-all BLASTP (*E*-value = 1 × 10^−5^). OrthoMCL was applied to process the BLASTP output and cluster genes to gene families. Similarity of protein families was set to be 0.5 as suggested by a previous publication^76^.

### Determination of gene presence or absence

We proposed a ‘map-to-pan’ strategy to determine gene presence or absence^28^. For the 453 accessions with high sequencing depth, although only about 60%–70% of their genomes can be de novo assembled (contig ≥ 500 bp), more than 98% of their genomes can be covered by short read mapping. This enabled the use of coverage of genes to determine their presence or absence. In practice, genes with CDS coverage over 0.95 and gene-body coverage over 0.85 were considered present. If one member of a gene family is present in a given rice accession, the gene family is considered as present.

### Determination of core and distributed genes or gene families

A core gene (or gene family) is a gene (or gene family) present in all rice accessions, and we further defined candidate core genes (or gene families) as those with loss rates not significantly larger than 0.01 in all major groups. We first examined whether a gene (or a gene family) is distributed (loss rate > 0.01) in each type of *O. sativa* (XI, GJ, cA and cB). Binomial tests (with a null hypothesis of loss rate < 0.01) were carried out for each gene in each type. A *P* value below 0.05 meant that this gene (or a gene family) was lost in a significant proportion of rice accessions and is a distributed gene (or gene family) of these subpopulations. If a gene (or a gene family) was not determined to be distributed in all types (and it was not core), it was considered to be a candidate core gene (or gene family) of *O. sativa*. Other genes (or gene families) were considered to be distributed.

### Determination of major-group-unbalanced, subpopulation-unbalanced and random genes or gene families

Distributed genes (or gene families) were divided further into major-group-unbalanced, subpopulation-unbalanced and random genes (or gene families). Major-group-unbalanced genes (or gene families) are defined as genes (or gene families) that are unequally distributed among XI, GJ, cA and cB groups. A two-sided Fisher’s exact test was used to determine whether the distribution of each gene (or gene family) is uniform. The *P* values of all genes were calculated with the ‘Fisher.test’ function in R and were then adjusted with the Benjamini–Hochberg FDR method. Genes (or gene families) with FDR < 0.05 were considered as major-group-unbalanced.

Subpopulation-unbalanced genes (or gene families) are defined as genes (or gene families) that are unequally distributed among subpopulations; thus, they can be divided into XI-subpopulation-unbalanced genes (or gene families) and GJ-subpopulation-unbalanced genes (or gene families). XI-subpopulation-unbalanced genes (or gene families) are defined as genes (or gene families) that are unequally distributed among XI-1A, XI-1B, XI-2 and XI-3 subpopulations. GJ-subpopulation-unbalanced genes (or gene families) can be defined similarly. The same statistical methods for the major groups were applied to determine the distribution balance for subpopulations. We defined genes (or gene families) that are neither major-group-unbalanced nor subpopulation-unbalanced to be ‘random’ genes.

### Gene and gene-family age

Gene ages were inferred with previously described methods^77^. The NR protein database was downloaded from NCBI (28 March 2015) and all protein sequences were grouped according to 13 taxonomic levels (PS1: Cellular organisms; PS2: Eukaryota; PS3: Viridiplantae; PS4: Streptophyta, Streptophytina; PS5: Embryophyta; PS6: Tracheophyta, Euphyllophyta; PS7: Spermatophyta; PS8: Magnoliophyta, Mesangiospermae; PS9: Liliopsida, Petrosaviidae, Commelinids, Poales; PS10: Poaceae; PS11: BOP clade; PS12: Oryzoideae, Oryzeae, *Oryza*; and PS13: *O. sativa*) based on NCBI taxonomy. Thirteen BLASTP databases were built for protein sequences from PS1 to PS13. All genes on pan-genome sequences were first translated into proteins, and were aligned to the 13 databases using BLASTP with *E*-values < 1 × 10^−5^ and identity > 0.3. The age of a gene was considered as the taxonomic level of the oldest aligned protein. Genes that failed to align to all databases were assigned gene ages of PS13 (*O. sativa*). Some PS13 genes were reassigned as PS12 genes if they could be covered by 446 wild rice genomes^2^ with both gene-body coverage > 0.95 and CDS coverage > 0.95. The age of a gene family was considered as the age of the oldest gene within the gene family.

### Introgression test

To test whether an XI sample had a non-introgression haplotype at a locus, we defined a *D*-value for a sample *x* as *D*(*x*) = *d*(*x*,XI) − *d*(*x*,GJ), in which *d*(*x*,XI) is the mean distance from sample *x* to a XI sample at the given locus.

With no gene flow from GJ to XI and vice versa, the *D*-value is negative for XI and positive for GJ. On the other hand, if an XI sample shares a haplotype with a GJ sample, the *D*-value will be positive and close to the *D*-values of GJ samples. For an XI sample, we rejected the hypothesis of GJ introgression if its *D*-value was negative and less than the lower bound of the 99% confidence interval for the *D*-value of GJ samples, which was computed on the subset of GJ consisting of samples with a positive *D*-value, to exclude the effect of potential XI-to-GJ introgression.

### Reporting summary

Further information on experimental design is available in the Nature Research Reporting Summary linked to this paper.

### Code availability

Code for studying pan-genome and gene and gene family PAVs are now integrated and published as the EUPAN toolkit^28^. Tailored novoBreak-germline is available at https://sourceforge.net/projects/novobreak/?source=navbar. Code for nucleotide diversity and SNP merging is available at https://github.com/dchebotarov/3k-SNP-paper. All other code is available from the corresponding authors upon request.

### Data availability

The BAM alignment file and variant calls in VCF format for each accession of the 3K-RG against Nipponbare RefSeq are freely downloadable from Amazon Public Data at https://aws.amazon.com/public-data-sets/3000-rice-genome/ and the Department of Science and Technology Advanced Science and Technology Institute of the Philippines (DOST-ASTI) IRODS site, as described on the 3K-RG project site (http://iric.irri.org/resources/3000-genomes-project). The SV and PAV data of 3K-RG are available in the figshare database^78^ (10.6084/m9.figshare.c.3876022.v1).

The following web tools are available for the mining, analysis and visualization of the 3K-RG dataset: SNP-Seek, http://snp-seek.irri.org; RMBreeding databases, http://www.rmbreeding.cn/index.php; rice cloud of genetic data public projects, http://www.ricecloud.org/; IRRI Galaxy, http://galaxy.irri.org/; and the 3,000 rice pan-genome browser^79^, http://cgm.sjtu.edu.cn/3kricedb/.

The 3K-RG sequencing data used for our analyses can be obtained via project accession PRJEB6180 from NCBI (https://www.ncbi.nlm.nih.gov/sra/?term=PRJEB6180), accession ERP005654 from DDBJ (https://www.ddbj.nig.ac.jp/index-e.html) and from the GigaScience Database (10.5524/200001).

## Online content

Any Methods, including any statements of data availability and Nature Research reporting summaries, along with any additional references and Source Data files, are available in the online version of the paper at 10.1038/s41586-018-0063-9.

## Supplementary information

Supplementary Notes This file contains Supplementary Notes and additional references – see contents page for details

Reporting Summary

Supplementary Data 1

Supplementary Data 2

Supplementary Data 3

Supplementary Data 4
