# Supplementary material for: Genomic variation in 3,010 diverse accessions of Asian cultivated rice
Source: Nature. 2018 Apr 25;557(7703):43–9. doi: 10.1038/s41586-018-0063-9 (PMC6784863; doi:10.1038/s41586-018-0063-9)
Supplement: Supplementary file 1 — This file contains Supplementary Notes and additional references – see contents page for details [file 41586_2018_63_MOESM1_ESM.pdf]

nature SUPPLEMENTARY INFORMATION
https://doi.org/10.1038/s41586-018-0063-9
In the format provided by the authors and unedited.

Genomic variation in 3,010 diverse accessions of Asian cultivated rice

Wensheng Wang[1,17], Ramil Mauleon[2,17], Zhiqiang Hu[1,3,17], Dmytro Chebotarov[2,17], Shuaishuai Tai[4,17], Zhichao Wu[1,5,17], Min Li[6,7,17], Tianqing Zheng[1,17], Roven Rommel Fuentes[2,17], Fan Zhang[1,17], Locedie Mansueto[2,17], Dario Copetti[2,8,17], Millicent Sanciangco[2], Kevin Christian Palis[2], Jianlong Xu[1,5,6], Chen Sun[3], Binying Fu[1,6], Hongliang Zhang[9], Yongming Gao[1,6], Xiuqin Zhao[1], Fei Shen[9], Xiao Cui[3], Hong Yu[10], Zichao Li[9], Miaolin Chen[3], Jeffrey Detras[2], Yongli Zhou[1,6], Xinyuan Zhang[5], Yue Zhao[3], Dave Kudrna[8], Chunchao Wang[1], Rui Li[3], Ben Jia[3], Jinyuan Lu[3], Xianchang He[3], Zhaotong Dong[3], Jiabao Xu[4], Yanhong Li[4], Miao Wang[4], Jianxin Shi[3], Jing Li[3], Dabing Zhang[3], Seunghee Lee[8], Wushu Hu[4], Alexander Poliakov[11], Inna Dubchak[11,12], Victor Jun Ulat[2], Frances Nikki Borja[2], John Robert Mendoza[13], Jauhar Ali[2], Jing Li[3], Qiang Gao[4], Yongchao Niu[4], Zhen Yue[4], Ma. Elizabeth B. Naredo[2], Jayson Talag[8], Xueqiang Wang[9], Jinjie Li[9], Xiaodong Fang[4], Ye Yin[4], Jean-Christophe Glaszmann[14,15], Jianwei Zhang[8], Jiayang Li[1,10], Ruaraidh Sackville Hamilton[2], Rod A. Wing[2,8]*, Jue Ruan[5]*, Gengyun Zhang[4,6]*, Chaochun Wei[3,16]*, Nickolai Alexandrov[2]*, Kenneth L. McNally[2]*, Zhikang Li[1,6]* & Hei Leung[2]

[1]Institute of Crop Sciences, Chinese Academy of Agricultural Sciences, Beijing, China. [2]International Rice Research Institute, Manila, Philippines. [3]School of Life Sciences and Biotechnology, Shanghai Jiao Tong University, Shanghai, China. [4]BGI Genomics, BGI-Shenzhen, Shenzhen, China. [5]Agricultural Genomics Institute, Chinese Academy of Agricultural Sciences, Shenzhen, China. [6]Shenzhen Institute for Innovative Breeding, Chinese Academy of Agricultural Sciences, Shenzhen, China. [7]Anhui Agricultural University, Hefei, China. [8]Arizona Genomics Institute, School of Plant Sciences, University of Arizona, Tucson, AZ, USA. [9]China Agricultural University, Beijing, China. [10]Institute of Genetics and Developmental Biology, Chinese Academy of Sciences, Beijing, China. [11]DOE Joint Genome Institute, Walnut Creek, CA, USA. [12]Lawrence Berkeley National Laboratory, Berkeley, CA, USA. [13]Advanced Science and Technology Institute, Department of Science and Technology, Quezon City, Philippines. [14]UMR AGAP, CIRAD, Montpellier, France. [15]UMR AGAP, Université de Montpellier, Montpellier, France. [16]Shanghai Center for Bioinformation Technology, Shanghai, China. [17]These authors contributed equally: Wensheng Wang, Ramil Mauleon, Zhiqiang Hu, Dmytro Chebotarov, Shuaishuai Tai, Zhichao Wu, Min Li, Tianqing Zheng, Roven Rommel Fuentes, Fan Zhang, Locedie Mansueto, Dario Copetti. *e-mail: lizhikang@caas.cn; k.mcnally@irri.org; ccwei@sjtu.edu.cn; ruanjue@caas.cn; nalexandrov@inariag.com; zhanggengyun@genomics.cn; rwing@ag.arizona.edu

© 2018 Macmillan Publishers Limited, part of Springer Nature. All rights reserved.

Genomic variation in 3,010 diverse accessions of Asian cultivated rice

Supplementary Notes

Wensheng Wang[1,17], Ramil Mauleon[2,17], Zhiqiang Hu[1,3,17], Dmytro Chebotarov[2,17], Shuaishuai Tai[4,17], Zhichao Wu[1,5,17], Min Li[6,7,17], Tianqing Zheng[1,17], Roven Rommel Fuentes[2,17], Fan Zhang[1,17], Locedie Mansueto[2,17], Dario Copetti[2,8,17], Millicent Sanciangco[2], Kevin Christian Palis[2], Jianlong Xu[1,5,6,] Chen Sun[3], Binying Fu[1,6], Hongliang Zhang[9], Yongming Gao[1,6], Xiuqin Zhao[1], Fei Shen[9], Xiao Cui[3], Hong Yu[10], Zichao Li[9], Miaolin Chen[3], Jeffrey Detras[2], Yongli Zhou[1,6], Xinyuan Zhang[5], Yue Zhao[3], Dave Kudrna[8], Chunchao Wang[1], Rui Li[3], Ben Jia[3], Jinyuan Lu[3], Xianchang He[3], Zhaotong Dong[3], Jiabao Xu[4], Yanhong Li[4], Miao Wang[4], Jianxin Shi[3], Jing Li[3], Dabing Zhang[3], Seunghee Lee[8], Wushu Hu[4], Alexander Poliakov[11], Inna Dubchak[11,12], Victor Jun Ulat[2], Frances Nikki Borja[2], John Robert Mendoza[13], Jauhar Ali[2], Jing Li[3], Qiang Gao[4], Yongchao Niu[4], Zhen Yue[4], Ma. Elizabeth B. Naredo[2], Jayson Talag[8], Xueqiang Wang[9], Jinjie Li[9], Xiaodong Fang[4], Ye Yin[4], Jean-Christophe Glaszmann[14,15], Jianwei Zhang[8], Jiayang Li[1,10], Ruaraidh Sackville Hamilton[2], Rod A. Wing[2,8], Jue Ruan[5], Gengyun Zhang[4,6], Chaochun Wei[3,16], Nickolai Alexandrov[2], Kenneth L. McNally[2], Zhikang Li[1,6] & Hei Leung[2]

[1]Institute of Crop Sciences, Chinese Academy of Agricultural Sciences, Beijing, China.
[2]International Rice Research Institute, Manila, Philippines
[3]School of Life Sciences and Biotechnology, Shanghai Jiao Tong University, Shanghai, China.
[4]BGI Genomics, BGI-Shenzhen, Shenzhen, China.
[5]Agricultural Genomics Institute, Chinese Academy of Agricultural Sciences, Shenzhen, China.
[6]Shenzhen Institute for Innovative Breeding, Chinese Academy of Agricultural Sciences, Shenzhen, China.
[7]Anhui Agricultural University, Hefei, China.
[8]Arizona Genomics Institute, School of Plant Sciences, University of Arizona, Tucson, AZ, USA.
[9]China Agricultural University, Beijing, China.
[10]Institute of Genetics and Developmental Biology, Chinese Academy of Sciences, Beijing, China.
[11]DOE Joint Genome Institute, Walnut Creek, CA, USA.
[12]Lawrence Berkeley National Laboratory, Berkeley, CA, USA.
[13]Advanced Science and Technology Institute, Department of Science and Technology, Quezon City, Philippines.
[14]CIRAD, UMR Agap, TA A-108/3, Montpellier, France.
[15]AGAP, University of Montpellier, CIRAD, INRA, Montpellier SupAgro, Montpellier, France.
[16]Shanghai Center for Bioinformation Technology, Shanghai, China

[17]These authors contributed equally: Wensheng Wang, Ramil Mauleon, Zhiqiang Hu, Dmytro Chebotarov, Shuaishuai Tai, Zhichao Wu, Min Li, Tianqing Zheng, Roven Rommel Fuentes, Fan Zhang, Locedie Mansueto, Dario Copetti.

Correspondence and requests for materials should be addressed to Zhikang Li (lizhikang@caas.cn), Kenneth L. McNally (k.mcnally@irri.org), Chaochun Wei (ccwei@sjtu.edu.cn), Jue Ruan (ruanjue@caas.cn), Nickolai Alexandrov nalexandrov@inariag.com), Gengyun Zhang (zhanggengyun@genomics.cn), and Rod A.Wing (rwing@ag.arizona.edu).

Contents

Overview of the sequencing quality of 3K rice data 2
Removal of 14 of the 3,024 accessions 3
k-mer analysis for genome characters 3
Mapping and genotype calling rate 3
Validation of SNP discovery pipeline 4
Validation of discovered SNPs in 3,010 accessions 5
Annotation of SNPs in the context of TEs 7
Inbreeding and heterozygous SNPs 8
SNP discovery and projection of undiscovered fractions 10
Utility of 3K RG for GWAS 12
Detection of genomic structural variations (SVs) 14
Correlation of presence/absence of SVs with plant heights 15
De novo assembly of 3,010 rice genomes 16
Evaluation of the quality of *de novo* assembly 16
Construction of the pan-genome sequences 17
Pseudogene detection 19
Length distribution of novel genes 19
Read mapping to the pan-genome 20
Evaluation of gene presence/absence detection 21
Estimating size of the rice pan-genome 23
The average gene/ gene family difference between two accessions 23
Phylogenetic analysis based on gene (or gene family) PAV 23
Inferring gene and gene family age with 446 wild rice genomes 24
References 25

Overview of the sequencing quality of 3K rice data

The methods of selecting the accessions for sequencing as well as the sequencing methodology (paired-end sequencing separated by ~450 bp on Illumina HiSeq2000) were described in detail in the 3,000 Rice Genomes data note[1]. Updated metadata information is available in **Supplementary Data 1 Table 1**.

Regarding data processing, paired-end reads were trimmed to 83 bp, generating 205,084,357,762 paired-end reads for 3,024 genomes. For 287 samples, two or more DNA libraries were created; whereas, for the other 2,737 samples, a single library was made. Usually, each sample library was sequenced on several flowcells. Every flowcell lane/sample library combination resulted in a pair of separate fastq files. The total number of fastq files was 51,060 (25,530 pairs). Most samples have 12 files (6 pairs sequenced independently). Most flowcell lanes contained 23 different samples that were separated by index sequences.

The sequencing depths of the 3,024 genomes ranged widely from 4x to 50x, with a mean of 14.3±6.3x, a median of 13.2x, and adjusted mean sequencing depth of 14.9±6.2x. Of these, 2,461 (81.4%) rice lines have sequencing depths over 10x and 458 (15.1%) have sequencing depths over 20x.

Before we carried out further analyses, we examined the quality of our sequencing data with the FastQC software (v0.11.2, http://www.bioinformatics.babraham.ac.uk/projects/fastqc/), with results available at http://oryzasnp.org/3kfastqc/.

Removal of 14 of the 3,024 accessions

Fourteen accessions were removed from the subsequent analyses. Samples CX400, CX401, CX402, IRIS_313-11415, and IRIS_313-10729 belong to African cultivated rice (*Oryza glaberrima* L.). Samples IRIS_313-8502, IRIS_313-9233, IRIS_313-8444, IRIS_313-10057, IRIS_313-9184, B014, and IRIS_313-9404 were removed due to significant contamination, and sample B101 was removed due to a very small estimated genome size. IRIS_313-8921 was removed because of its extremely low sequencing depth (~0.3x).

k-mer analysis for genome characters

We used the 17 bp *k*-mer analysis with *k*-mer_count (in-house software, written by Jue Ruan) to calculate the distribution of *k*-mer frequency for genome character estimation (genome size, repeat ratio and GC content) with total reads of each accession. The calculation is based only on the statistic of *k*-mers over the first trough point of the 17 bp *k*-mer frequency distribution graph; the data before that were considered to be sequencing errors. The genome size (GS) = total number of *k*-mers /peak value of *k*-mers frequency, repeat ratio = (GS-number of *k*-mers with different sequence + number of *k*-mers before depth of the first trough point)/GS.

Mapping and genotype calling rate

The alignment statistics table (**Supplementary Data 2 Table 1**) shows that the highest mapping rate of reads for an accession occurs when the accession and

reference genome belong to the same variety group (grouping is determined by population analysis described in subsequent sections), which also indicates that more genotyping calls are detected when the accession and reference genome belong to the same variety group. It is also notable that there is no appreciable increase in genotyping sensitivity from sequencing coverage 30x and beyond (**Supplementary Data 2 Figure 1**).

Validation of SNP discovery pipeline

To validate the GATK UnifiedGenotyper (GATK-UG)[2] SNP discovery pipeline, we compared the SNPs discovered by this pipeline against a standard SNP discovery method on two selected subset accessions. For the standard method, we used the MUMmer[3] pipeline to first discover SNPs between two published reference genomes, Nipponbare RefSeq (Nipponbare IRGSP 1.0 genome) and IR 64 (os.ir64.cshl.draft.1.0). Reciprocal whole-genome alignment was carried out using nucmer using default settings. SNPs from this alignment were extracted from each reciprocal alignment using show-snps with the parameters "-C -l -r -T". The common SNPs from each reciprocal alignment (same positions, same allele states) were extracted and 776,622 high-quality SNPs were discovered between the Nipponbare and IR64 references (which we call the Nipponbare-IR 64 reference SNPs).

We then selected the two re-sequenced accessions, CX140 (Nipponbare) and CX403 (IR 64a), from the 3K RG that have the same name but were of different provenance as the reference/published genomes. SNPs were discovered for these

accessions for IR 64 ref vs. CX140, and Nipponbare ref vs. CX403, using the GATK-UG pipeline with high-quality SNPs selected by strict filtering criteria (not flagged as LowQual, no missing alleles, homozygous only). Of the 689,797 Nipponbare ref:CX403 and 699,429 IR 64 ref:CX140 SNPs that shared common positions with the Nipponbare-IR 64 reference SNPs, 99.9% of the alternate allele calls were concordant, indicating that the GATK-UG pipeline performed well in SNP calling.

Validation of discovered SNPs in 3,010 accessions

The ~27 million biallelic SNPs from 3K RG were also compared with those discovered by previous projects: (1) the 44k SNP project[4], (2) the Rice50 SNPs project[5], (3) SNP-Seek release 1[6], and (4) dbSNP rice release 147[7] (https://www.ncbi.nlm.nih.gov/projects/SNP/index.html).

Using 44,100 SNPs from the 44k SNP project, most (36,775) of the original 44k SNPs could be re-mapped to the Nipponbare RefSeq. Of the re-mapped 44k SNPs, 94.5% are in the same position as the 3K RG biallelic SNPs, and in this common set, 99.8% have the same alternate allele calls.

SNPs from the 50 resequenced rice genomes[5] (6,496,456 high-quality Rice50 SNPs) were compared for concordance with the 3K RG SNPs. Since the Rice50 SNPs were anchored to the IRGSP4 Nipponbare genome assembly, these were mapped to the Nipponbare RefSeq, and the overwhelming majority (>99.9%) of the SNPs (6,496,018) from Rice50 were anchored to this reference version. The intersection

with the 3K RG SNPs showed that ~56% of the Rice50 SNPs (3,669,353) mapped to the same position as the 3K RG SNPs. However, since many of these Rice50 SNPs had ambiguous or heterozygous alternate allele calls (1,559,842), these were excluded in the concordance comparison, leaving 2,109,511 common SNP positions having definite alternate calls. For these common SNP positions, ~2.09 million were concordant in alternate allele calls (99.1%).

The full 3K RG Nipponbare SNP dataset (~29 million SNPs) was contrasted to the ~20.3 million SNPs in the first release[6] (2015) of SNP-Seek to determine the effect of earlier software versions of BWA (ALN vs MEM)[8] and GATK (2 vs 3)[2] on SNP discovery. Although the majority of the SNPs in the new set are common to those in the 2015 SNP-Seek release (~17.54 million, 86% of the release 1 set), ~14.5 million SNPs are unique to the new 3K RG SNPs (45.3% of the release 2 set) and ~2.77 million SNPs (13.7% of the release 1 set) are unique to the 2014 SNP-Seek release, highlighting the effects of using updated software on variant detection. Therefore, it is worthwhile to update SNP discovery analyses as newer (and better) SNP calling software becomes available.

We also compared the ~27 million biallelic SNPs from 3K RG with the NCBI Reference cluster ID (rs#) SNPs from chromosomes 1 to 12 of build 147 of dbSNP - rice (9,922,318 SNPs and 230,253 small indels of <=50 bp). All rs# SNPs were re-mapped (using the flanking sequence information) to the current build of the Nipponbare RefSeq prior to comparison using mega-BLAST of NCBI, and alignments where the entire flanking sequence aligns with one mismatch on the SNP

position were selected for the SNP comparison. A total of ~8.5 million rice SNPs in dbSNP remapped to Nipponbare RefSeq, and 51.5% of the remapped dbSNPs had common positions with the 3K RG SNPs. Of this common position SNP set, 4,199,667 remapped dbSNPs and 3K RG SNPs had the same alternate allele call (96% concordance). In all, ~4.3 million remapped rice dbSNPs are unique from the 3K RG set, and 25.4 million 3K RG SNPs are unique from rice dbSNPs. In comparison with the newest submitted NCBI Assay ID (ss#) SNPs in dbSNP 147 (Q4 2014 submission by McCouch et al.[9], 700,000 SNPs with ss#), there were relatively higher numbers of intersecting and concordant SNPs with the 3K RG SNPs (538,887 of 700k intersecting the 3K RG SNPs at 99% concordance).

Annotation of SNPs in the context of TEs

The repeat content of the Nipponbare RefSeq is 48% of the total genome, with most of the discovered SNPs occurring in LTR retro-elements and TIR DNA transposon repeat types, the bulk of repeats in the rice genome. Repeats associated with telomeres and centromeres, although comprising a very small portion of the genome, exhibit the highest SNP densities observed (163 and 177 SNPs/kb); however, the number of SNPs in these repeat types is too low to impact the number of SNPs discovered. The average genome-wide SNP density (combined repetitive and non-repetitive regions) is lower (72 SNPs/kb) than the average SNP density in repetitive regions only (103 SNPs/kb) and even lower in the non-repeat portion of the genome (44 SNPs/kb) (**Supplementary Data 2**).

Inbreeding and heterozygous SNPs

Given that (a) rice is a selfing species, (b) accessions were subjected to single seed descent before sequencing, and (c) there is a high degree of differentiation between groups of rice varieties, one thus expects the number of heterozygous calls per SNP to be much lower than postulated by Hardy-Weinberg equilibrium. Indeed, we observe this systematic deviation from HW equilibrium in the 3K dataset (**Extended Data Fig.1a**) as well as in major subpopulations.

To facilitate the discussion, let us introduce the following notation:

Hobs: observed SNP heterozygosity, the proportion of heterozygous calls in all non-missing calls of a SNP.

Hexp: expected SNP heterozygosity given by Hardy-Weinberg equilibrium, i.e. "Hexp=2pq", where p and q are the two allele frequencies.

The ratio Hobs/Hexp is expected to be distributed around 1-F, where F is Wright's inbreeding coefficient. We use the distribution of Hobs/Hexp to estimate F and remove outlier SNPs that might represent alignment errors.

We analyzed Hobs/Hexp distribution in the whole 3K SNP dataset, as well as in two subsets: XI (1,789 samples) and GJ (772 samples). In all three datasets, the distribution of Hobs/Hexp is bimodal, with one peak harboring most of the common SNPs and that corresponds to a higher F (~0.95) and the other peak at around "Hobs/Hexp=1" caused mostly by rare SNPs with low numbers of homozygous

alternate calls and an excess of heterozygotes.

There is an excess number of points along the upper left boundary of the scatterplot (**Extended Data Fig. 1a**) that corresponds to a maximal value of "Hobs=2p", where p is the minor allele frequency, indicating an excess of SNPs with no or very few homozygous alternate calls. We hypothesize that these SNPs are due to alignment errors caused by duplications that do not occur in a reference but are present in certain genotypes.

We estimate the inbreeding coefficient F for XI and GJ datasets, as well as (effective) inbreeding coefficient in the whole 3K dataset as the median value of "1-Hobs/Hexp" for SNPs where "Hobs/Hexp <1" and the minor allele frequency is >5%. Doing so, we ignore the peak near "Hobs/Hexp =1" as it is likely an alignment artifact where reads map to multiple regions that are duplicates. The resulting estimates are F=0.954 for the whole 3K, F=0.925 for XI, and F=0.969 for GJ.

We use the estimates of F to introduce a quality cutoff for number of observed heterozygotes:

$$Hobs\_max = 10\ (1-F)\ Hexp$$

That is, a SNP whose heterozygosity is >10x higher than the most likely value for a given frequency and the dataset's inbreeding rate will be deemed as having an excessive number of heterozygotes and will be filtered out. The cutoff values for different datasets are thus 0.4795082 for 3K, 0.7485704 for XI, and 0.3106786 for GJ datasets.

We remove SNPs that violate this quality criterion in the 3K XI and GJ datasets from the set of biallelic SNPs (**Extended Data Fig.1b**). We call the resulting set of 16,874,733 SNPs polymorphic in 3010 genomes as the **Base SNP set**.

Of note is that, although we expect that these might be erroneous calls, the fact they occur preferentially in the third base of codons and exhibit properties for coarse classification indicates that there is some biological significance meriting further investigation.

SNP discovery and projection of undiscovered fractions

Estimated proportion of IRRI Genebank SNPs discovered in 3,010 samples for a given allele frequency.

For a SNP that has a frequency f in the genebank, the probability that it has been observed in 3,010 samples can be approximated by

$$\text{Prob (observed in 3K)} = 1 - (1-f)^{3010}$$

Note that this approximation is robust due to the large size of the genebank; using the exact hypergeometric formula gives a probability estimate that differs from this by at most 0.4% at any point. Note also that this is a conservative estimate, since we treat each sample as haploid when in reality each is diploid.

Using this function, one can estimate that 3,010 samples capture more than 99.9% of genebank SNPs of frequency greater than 0.25%, and virtually 100% of SNPs of frequency >1.1% (**Extended Data Fig. 1c**).

However, this function alone does not allow estimation of the proportion of the

total number of SNPs (of all frequencies) captured by the 3K, since the distribution of SNP frequencies is not known. This distribution depends on past demographic events and selection. To estimate the total number of undiscovered SNPs, we adopted the simulation approach outlined below.

Estimation of the total number of Nipponbare RefSeq-based SNPs occurring in genebank samples

We computed SNP discovery rates based on 6,000 random permutations of samples from the **Base** SNP set in the following way. For each permutation of sample order, we computed the number of new SNPs added by each consecutive sample (i.e. SNPs not seen in previous samples either as HOM or as HET). We then computed the mean number of additional SNPs when an Nth sample is added across all permutations and projected the mean for the range [3,010, 120,000] using regression between log(mean_new_SNP) and log(sample).

The fitted model is

$$\log(\text{mean\_new\_SNP}) = -0.75*\log(\text{sample}) + 5.74$$

with $R^2 = 0.995$.

Then, we estimated the number of SNPs as a sum of the estimated mean added SNPs at each value from 3,009 to 120,000.

As a result, we find that ~27M new SNPs are estimated to be discovered upon genotyping the rest of the genebank, leading to ~44M total Nipponbare-based SNPs.

Extended Data Fig. 1d shows the number of SNPs added with each sample, on a log-log scale, with a linear regression fit. One can also see from this graph, if the

trend continues as shown, it would be possible to extrapolate the number of SNPs that may occur in the entire population of *O. sativa* including those not yet conserved in genebank(s). This analysis is based on a subset of the **Base** SNP set consisting of 3,006 samples (in addition to the previously identified problematic samples, we removed 4 samples that were outliers in terms of many private SNPs and made extrapolation harder).

Similar analyses were done for XI and GJ separately, estimating 14M and 13M new SNPs, respectively. However, one needs to model the overlap, and there is some sharing of even the rarest non-singleton SNPs. Since this makes the modeling unduly complicated, we report only the analysis based on the whole set.

Utility of 3K RG for GWAS

As a test case, a genome-wide association study (GWAS) was conducted using historical phenotypic data for grain length (GRLT) and grain width (GRWD) and newly acquired data for bacterial blight (BLB) resistance, with an LD pruned subset of the 3K RG.

1. Sample and SNP filtering

We performed quality control measures by filtering low quality samples and markers for use in GWAS. The samples were filtered to remove those for which missing data or call rates (CR) were below 80% and with an over- and under-abundance of heterozygous SNPs in the interquartile range (IQR), calculated as the difference between the upper and lower quartiles (IQR = Q3 – Q1). We also excluded markers

with an over-abundance of heterozygous alleles (number of alleles >2), and those markers with low call rates (CR <0.9) and minor allele frequencies (MAF <0.05). Further, we pruned the dataset based on LD using the Composite Haplotype Method (CHM)[10] algorithm, with the following parameters: window size of 35 SNPs, window increment of 15 SNPs, and r^2 threshold of 0.5. The LD pruned datasets had 2,012 samples and 223,743 markers for GRLT and GRWD and 381 samples and 148,999 markers for BLB.

2. Phenotypic data

We performed GWAS for source accessions of the sequenced genetic stocks with historical phenotypic data for grain length (GRLT) and grain width (GRWD) and newly-quantified bacterial leaf blight (BLB) scores. Trait data for GRLT and GRWD were collected from unreplicated trials as genebank characterization data over many seasons of trials; data for the source accession of the sequenced genetic stock were used as a proxy for those of the derived genetic stock that underwent one or more cycles of single seed descent from the source accession. BLB resistance was measured as lesion length after infection with C5 Chinese strain of *Xanthomonas oryzae* on the genetic stocks that were used for sequencing.

3. GWAS methods

We implemented an EMMAX (Efficient Mixed-Model Association eXpedited)[11] single-locus mixed linear model in SNP & Variation Suite v8.4.0 software (http://www.goldenhelix.com) for GWAS. EMMAX allows correction for cryptic relatedness and other fixed effects using a kinship matrix (as random effect) and

population stratification using the top four principal components (as fixed effect). Both the kinship matrix and the principal components were generated from the LD pruned datasets. We used the False Discovery Rate (FDR <0.01) multiple testing correction to identify significant markers, and generated Manhattan and QQ plots from the EMMAX output using the qqman package[12] in R.

Detection of genomic structural variations (SVs)

We tested BreakDancer[13], DELLY[14], and novoBreak[15] (https://sourceforge.net/projects/novobreak/?source=navbar) for SV calling against the Nipponbare RefSeq and with several SVs inserted into the Nipponbare genome (**Supplementary Data 3 Tables 5 and 6**). novoBreak was found to have the lowest false positive rate and comparatively good resolution and was therefore selected for all subsequent SV detection. Briefly, novoBreak employed a *k*-mer (contiguous nucleotide sequence of length k) targeted local assembly algorithm to detect structural variation breakpoints in single base pair resolution. When applied to the 3K RGs for discovering SVs, novoBreak first constructed a hash table of all the reads of a sample. Next, any *k*-mers matching the Nipponbare RefSeq were removed. Then, novoBreak employed a counting bloom filter to calculate the occurrence of all *k*-mers. At this step, low-frequency *k*-mers reflecting sequencing errors were removed. Next, high-frequency *k*-mers and their associated read pairs were clustered by a modified union-find algorithm, ensuring that each cluster represented a single breakpoint. Then, for each cluster, an assembler with a greedy algorithm was applied to assemble the

read pairs spanning the breakpoint into optimal and sub-optimal contigs. By comparing the assembled contigs with the Nipponbare RefSeq, novoBreak inferred the exact breakpoints of all types of SVs. Finally, novoBreak scored each SV based on alignment and assembly evidence and a filter was applied to generate a high-confidence SV list. In novoBreak, the detected translocations were referred to as 'inter-chromosomal breakpoints'. We detected SVs in the 3,010 accessions. In order to minimize the probability of false positives, SVs detected in fewer than 6 accessions (the number of such SVs = 207,879) or in more than 80% (the number of such SVs = 446) of the 3,010 accessions were removed. We analyzed the SVs detected in 453 well-sequenced accessions. Translocations and deletions account for 74.3% and 21.4% of all SVs, respectively. Inversions and duplications account for only 1.7% and 2.4%, respectively. The percentage of SVs detected may reflect both the real number of different type SVs in the genomes and the false positives and negatives in SV detection. Genes interrupted by SVs or inside SV regions were identified and we also checked the co-localization between TEs and SVs (**Supplementary Data 3 Table 7**), with 1 kb, 5 kb, and 10 kb windows with one breakpoint as the background. TE annotation was from RGAP 7[16].

Correlation of presence/absence of SVs with plant heights

This was calculated using 'cor.test' (method="spearman") in R. The SV with the highest correlation was a ~385 bp deletion, located in the *sd1*[17] gene (LOC_Os01g66100) (rho= -0.40, *P*-value = 2.48E-10). The average height of

accessions with the deletion was 84.96 cm, while it was 126.50 cm for accessions without the deletion.

De novo assembly of 3,010 rice genomes

In order to gain better assemblies, we compared the performance of several assembly tools developed for NGS including SOAPdenovo version r240[18], Velvet version 2.2.5[19] and SPAdes version 3.0.0[20]. Finally, a method with iterative use of SOAPdenovo was selected for the 3K rice assembly that had better performance than SPAdes and relatively good speed (~3.94 times running time of default SOAPdenovo). The key idea of this variant method was to select the best *k*-mer for each sample.

QUAST version 2.3[21] was used for evaluation of the assemblies, including 1) comparison of assemblies among SOAPdenovo, Velvet and SPAdes and 2) comparison among all rice accessions with our variant method described above with parameter "-t 16 --min-contig 500 -o output --no-plots -R IRGSP-1.0.fa". The Nipponbare RefSeq (IRGSP-1.0) genome was used for all the evaluations. The Nipponbare RefSeq was downloaded from the Rice Annotation Project (RAP)[22]. Several important indices, including N50, assembled size, genome fraction (how much of the Nipponbare RefSeq can be covered with the assembled contigs), and unaligned contig size, were selected to evaluate the assembly performance.

Evaluation of the quality of *de novo* assembly

In order to evaluate the quality of *de novo* assembly, we first developed a pipeline to

correct/remove the possible misassembled contigs from read mapping. Reads (used for the assembly) are mapped to the assembled contigs and we broke down the contigs at positions with no evidence of connections supported by read alignments. Remaining fragments shorter than 500 bp were removed.

Next, we assessed the assembly results based on accessions CX140 (compared with the Nipponbare RefSeq), CX133 (compared with the Zhenshan 97 genome[23]), and CX145 (compared with the Minghui 63 genome[23]). For CX140, we first applied the correction procedure to both raw SOAPdenovo contigs and GapCloser contigs. For CX145 and CX133, only raw SOAPdenovo contigs were evaluated. The assemblies were assessed by QUAST with the Nipponbare RefSeq as a gold standard. Results are shown in **Supplementary Data 3 Tables 8 and 9.**

The mis-assembly rate of SOAPdenovo is quite low (~0.1%). Obviously, GapCloser improved the assembly indices dramatically, including total length, N50, and genome fraction. However, it introduced >20 times the amount of mis-assemblies. A large part of these mis-assemblies can be corrected, but a significant proportion still remained. Therefore, we decided to remove the GapCloser step for construction of the pan-genome sequence. In addition, mis-assemblies in the SOAPdenovo results could not be removed by the correction procedure, indicating that most of them are reliable assembled contigs. Hence, we concluded that assembly errors should be very low.

Construction of the pan-genome sequences

We constructed the pan-genome of rice with the Nipponbare RefSeq and

non-redundant novel sequences in the assembly of 3,010 rice accessions. The Nipponbare RefSeq was selected because (1) this genome has relatively good annotation and (2) it is widely used for current rice studies, which enables our pan-genome outcome to be integrated easily with current and historical rice studies that have employed the same, giving researchers the ability to easily identify presence/absence of their gene(s) of interest in the rice accessions involved in their study, as well as the attributes of these genes (core, distributed, or GJ-specific, etc.).

Blast was used to evaluate the pan-genome. The global identity (G_{iden}) of contig C was calculated as follow:

$$G_{iden}(C) = \sum_{i=1}^{N} w_i \cdot M_i \cdot P_i / L_c$$

$$w_i = \begin{cases} 1, HSP_i \text{ doesn't overlap with any of } HSP_1, \dots, HSP_{i-1}; \\ 0, HSP_i \text{ overlaps with at least one of } HSP_1, \dots, HSP_{i-1}; \end{cases}$$

where M_i is length of the HSP_i; P_i is the percent identity of HSP_i; L_C is the length of the contig C; and w_i is a weight indicating whether HSP_i overlaps with previous HSPs. Using this method, we can retrieve novel sequences with an identity cutoff at any value (0.3, 0.5, or 0.7, etc.) in comparison to the Nipponbare RefSeq. Similar methods were used to remove redundant sequences. Like CD-HIT, we use a 'longest sequence first' list removal algorithm to remove sequences above a given identity (see CD-HIT software for details). The difference is that we calculate the global identity of two contigs (A and B) based on NCBI-blast similar to the above method:

$$G_{iden}(A, B) = G_{iden}(B, A) = \sum_{i=1}^{N} w_i \cdot M_i \cdot P_i / \min(L_A, L_B)$$

$$w_i = \begin{cases} 1, HSP_i \text{ doesn't overlap with any of } HSP_1, \dots, HSP_{i-1}; \\ 0, HSP_i \text{ overlaps with at least one of } HSP_1, \dots, HSP_{i-1}; \end{cases}$$

where M_i is length of the HSP_i; P_i is the percent identity of HSP_i; L_A and L_B are the lengths of the contigs *A* and *B*; and w_i is a weight indicating whether HSP_i overlaps with previous HSPs. The longest contig within each cluster is selected as the representative. Combining these two steps, we can retrieve non-redundant novel sequences at any identity cutoff of P (these sequences have global identities below P in comparison with IRGSP genome sequences, as well as among the sequences themselves).

Pseudogene detection

The sequences of predicted single-exon genes from the novel sequences were extracted and aligned to all other gene sequences using NCBI-blastn with E-value = 1e-5. A novel single-exon gene with >90% of its full length similar to another gene (a multi-exon novel gene or a Nipponbare gene) is defined as a candidate pseudogene. As a result, 1,030 of the 12,465 genes might be candidate pseudogenes.

Length distribution of novel genes

We checked the gene length differences of the novel genes by comparing them with Minghui 63 (MH63)/Zhenshan 97 (ZS97) genes[23]. The MH63 or ZS97 protein sequences (for only the longest ORF of a gene with alternative transcripts removed) were first aligned to all pan-genome proteins; then, each MH63 or ZS97 protein with

its best hit (measured by E-value and global identity defined as "2*aligned_length/(query_length+target_length)") was considered as a pair. Those pairs with the pan-genome proteins in multiple pairs were further removed, forming a "single-copy" gene pair set. This set includes 2,474 MH63 genes and 2,441 ZS97 genes, each of which formed a pair with its corresponding novel gene; then, the length ratio was calculated and the density was plotted using a log2 scale (**Extended Data Fig. 5**). Generally, the distribution should be symmetric: ratio >0 means the novel gene is longer while a ratio <0 means the novel gene is shorter.

Read mapping to the pan-genome

Mapping raw reads of all rice accessions to the pan-genome sequences is an essential step in our pan-genome analysis. With the mapping results, we can (1) evaluate the sequencing quality based on the percentage of mapped reads and the comparison of sequencing and mapping depths; (2) determine presence/absence of pan-genome contigs in each rice accession; and (3) determine presence/absence of each gene in each rice accession.

We compared several mapping tools including SOAP version 2.21[24], Bowtie2 version 2.2.3[25], and BWA version 0.7.10[8] (both 'bwa aln' usage and 'bwa mem' usage) based on simulating reads from the 93-11 genome (http://rice.genomics.org.cn/rice2/link/download.jsp) and mapping reads to the Nipponbare RefSeq. A gold standard of alignments of the 93-11 genome and Nipponbare RefSeq was built with MUMmer[3]. Finally, 'bwa mem' was selected for

all genomic mapping tasks in our pan-genome analysis. The mapping depth and mapping coverage for the Nipponbare RefSeq of each rice line were calculated with Qualimap version 2.0[26] and bamUtil (https://github.com/statgen/bamUtil), respectively.

Evaluation of gene presence/absence detection

Gene presence/absence detection is a necessary step in high-resolution pan-genome analyses. Previous pan-genome studies in bacteria and rice assembled and annotated each individual genome separately. Here, we compared our method to a recent rice pan-genome study with three representative rice accessions, including the Nipponbare RefSeq[27]. In their study, they assembled the genomes from deep sequencing and multiple sequencing libraries. They were able to assemble 81.3~82.5% of non-N bases of each genome (81.8% for Nipponbare). This number increased to 88.5~91.4% (91.4% for Nipponbare) if Ns are considered, and they predicted 39,083 genes for the Nipponbare genome. Therefore, the total gene number should be 42,852~47,779 if the entire genomes are assembled and the gene density remains the same (actually, there should be fewer genes in the remaining sequences, which are mostly composed of repetitive and low-complexity sequences). Nevertheless, we can estimate the sensitivity and specificity of gene presence-absence detection approximately. The gold standard annotation for the Nipponbare RefSeq has 35,633 annotated genes. Assuming that all genes on the assembled sequences are correctly predicted (as we also used reference annotations directly in our analyses), the sensitivity can be

estimated (as the assembled fraction of the genome) to be about 81.3% or 91.4%. If all the reference genes can be predicted, the specificity should be 35,633/(>39,083/0.914) = 83.1%.

We then evaluated the accuracy of our method based on the result of CX140 (an independent accession of Nipponbare with sequencing depth at 19x). We detected 41,039 genes present in the CX140 *de novo* assembly, including 34,759 reference genes and 6,280 novel genes. The sensitivity is 34,759/35,633=97.5%. The mapping coverage of the CX140 genome is 98.4%; therefore, we think we can capture almost all genes for which mapping evidence exists. The specificity can be estimated as 34,759/41,039=84.7%, which is higher than with traditional pan-genome methods[27].

However, there are still a significant number of falsely discovered genes. In-depth studies suggest that all of these gene regions show high similarity (>90%) to the reference genome, indicating that these corresponding regions in the reference genome contained no genes, but that we are able to predict genes on similar sequences. Such gene calls might be the false positives from gene predictions, as in the previous work[27] in which 39,083 genes were predicted on the incomplete sequences, ~3,000 genes more than the RAP annotation[22]. Alternatively, this might be partially attributed to gene loss in the reference due to SNPs and small indels. This might be a short-coming of our mapping-based pan-genome study. Nevertheless, these false positives are still based on gene sequences and are not random, and sequences of the genes are indeed present in the genome. We therefore concluded that our mapping-based method has relatively good accuracy with very high sensitivity and

reasonable specificity.

Estimating size of the rice pan-genome

The sizes of the pan-genome, core gene families, and candidate core gene families were estimated based on simulations. We randomized the order of the 453 rice accessions for 500 times. Each time, we counted the number of core gene families and pan-genome for the first i accessions (i = 1, 2, …, 453) based on the predefined order. **Fig. 4c** shows the simulation results. The lighter lines stand for the results from 500 times randomization and the dark lines stand for the mean values. This showed that the total number of gene families of the rice pan-genome stabilized when the number of accessions was larger than 100.

The average gene/ gene family difference between two accessions

The average gene / gene family difference between two accessions (**Fig. 4e** and **Extended Data Fig.7e**) were calculated as the average of all combinations of each 2 of 453 accessions. The average proportions were calculated as the number of such differentiating gene families adjusted by the average gene / gene family numbers held in common by the two major groups.

Phylogenetic analysis based on gene (or gene family) PAV

The core genes (or gene families) present in all rice accessions by definition provide no variation. Only the distributed genes (or gene families) were used for the

phylogenetic study. The gene (or gene family) presence/absence information of the 453 rice accessions was arranged as a 0-1 matrix with each line representing a gene (or gene family) and each column representing a rice accession. The PARS program within PHYLIP (http://evolution.genetics.washington.edu/phylip.html) was used to infer the phylogenetic relationship from the presence/absence matrix. The phylogenetic tree was subsequently plotted with the APE[28] package in R.

Inferring gene and gene family age with 446 wild rice genomes

Most of the more than 10,000 novel genes were assigned an age of PS13. It is unlikely that such a large number of genes arose within less than the 10,000 years of rice domestication. We therefore inferred that these genes already existed in the wild progenitors of rice; to check this point, we interrogated the whole-genome sequencing data of 446 wild rice accessions[29]. In Huang et al.'s paper[29], 446 wild accessions were previously classified into three groups: Or-I (OR-XL), Or-II (OR-Int), and Or-III (OR-GL). We used the "map-to-pan" strategy[30] to study if *O. sativa* genes exist in Or-Int, Or-XL, and Or-GL. To overcome the shortcoming of insufficient sequencing depth of each accession, sequencing data of the same group (Or-Int, Or-XL and Or-GL) were merged and then mapped to the pan-genome sequences of *O. sativa* L., and genes with both gene body coverage >0.95 and CDS coverage >0.95 were considered as present. As a result, we found that 98.95% of *O. sativa* genes could be detected in wild rice, including >99.9% of the core genes and 96.9% of the distributed genes. Moreover, 437 of the 528 XI-private genes, 110 of the 132 GJ-private genes,

56 of the 61 cA-private genes, and 41 of the 48 cB-private genes could be detected in wild rice. Genes found in wild rice that were previously labeled as PS13 were assigned an age of PS12.

References

1 The 3K RGP. The 3,000 rice genomes project. *GigaScience* **3**, 1-6 (2014).

2 McKenna, A. *et al.* The Genome Analysis Toolkit: a MapReduce framework for analyzing next-generation DNA sequencing data. *Genome Res.* **20**, 1297-1303 (2010).

3 Delcher, A. L., Salzberg, S. L. & Phillippy, A. M. Using MUMmer to identify similar regions in large sequence sets. *Curr. Protoc. Bioinf.* 10.13. 11-10.13. 18 (2003).

4 Zhao, K. *et al.* Genome-wide association mapping reveals a rich genetic architecture of complex traits in Oryza sativa. *Nat. Commun.* **2**, 467 (2011).

5 Xu, X. *et al.* Resequencing 50 accessions of cultivated and wild rice yields markers for identifying agronomically important genes. *Nat. Biotechnol.* **30**, 105-111 (2012).

6 Alexandrov, N. *et al.* SNP-Seek database of SNPs derived from 3000 rice genomes. *Nucleic Acids Res.* **43**, D1023 (2015).

7 Sherry, S. T. *et al.* dbSNP: the NCBI database of genetic variation. *Nucleic Acids Res.* **29**, 308 (2000).

8 Li, H. & Durbin, R. Fast and accurate short read alignment with Burrows–Wheeler transform. *Bioinformatics* **25**, 1754-1760 (2009).

9 McCouch, S.R. *et al*. Open access resources for genome-wide association mapping in rice. *Nat. Commun*. **7**, 17532 (2016).

10 Zaykin, D. V., Meng, Z. & Ehm, M. G. Contrasting linkage-disequilibrium patterns between cases and controls as a novel association-mapping method. *Am. J. Hum. Genet.* **78**, 737-746 (2006).

11 Kang, H. M. *et al.* Variance component model to account for sample structure in genome-wide association studies. *Nat. Genet.* **42**, 348-354 (2010).

12 Turner, S. qqman: QQ and manhattan plots for GWAS data. *R package version 0.1* **2** (2014).

13 Chen, K. *et al.* BreakDancer: an algorithm for high-resolution mapping of genomic structural variation. *Nat. Methods* **6**, 677-681 (2009).

14 Rausch, T. *et al.* DELLY: structural variant discovery by integrated paired-end and split-read analysis. *Bioinformatics* **28**, i333-i339 (2012).

15 Chong, Z. *et al.* novoBreak: local assembly for breakpoint detection in cancer genomes. *Nat. Methods* **14**, 65-67 (2017).

16 Kawahara, Y. *et al.* Improvement of the *Oryza sativa* Nipponbare reference genome using next generation sequence and optical map data. *Rice* **6**, 4 (2013).

17 Sasaki, A. *et al.* Green revolution: a mutant gibberellin-synthesis gene in rice. *Nature* **416**, 701-702 (2002).

18 Luo, R. *et al.* SOAPdenovo2: an empirically improved memory-efficient short-read de novo assembler. *GigaScience* **1**, 18 (2012).

19 Zerbino, D. R. & Birney, E. Velvet: algorithms for de novo short read assembly using de Bruijn graphs. *Genome Res.* **18**, 821-829 (2008).

20 Bankevich, A. *et al.* SPAdes: a new genome assembly algorithm and its applications to single-cell sequencing. *J. Comput. Biol.* **19**, 455-477 (2012).

21 Gurevich, A., Saveliev, V., Vyahhi, N. & Tesler, G. QUAST: quality assessment tool for genome assemblies. *Bioinformatics* **29**, 1072-1075 (2013).

22 Ohyanagi, H. *et al.* The Rice Annotation Project Database (RAP-DB): hub for *Oryza sativa* ssp. japonica genome information. *Nucleic Acids Res.* **34**, D741-D744 (2006).

23 Zhang, J. *et al.* Extensive sequence divergence between the reference genomes of two elite *indica* rice varieties Zhenshan 97 and Minghui 63. *Proc. Natl. Acad. Sci. USA* **113**, E5163-E5171 (2016).

24 Li, R. *et al.* SOAP2: an improved ultrafast tool for short read alignment. *Bioinformatics* **25**, 1966-1967 (2009).

25 Langmead, B. & Salzberg, S. L. Fast gapped-read alignment with Bowtie 2. *Nat. Methods* **9**, 357-359 (2012).

26 García-Alcalde, F. *et al.* Qualimap: evaluating next-generation sequencing alignment data. *Bioinformatics* **28**, 2678-2679 (2012).

27 Schatz, M. C. *et al.* Whole genome de novo assemblies of three divergent strains of rice, *Oryza sativa*, document novel gene space of aus and indica. *Genome Biol.* **15**, 506 (2014).

28 Paradis, E., Claude, J. & Strimmer, K. APE: analyses of phylogenetics and evolution in R language. *Bioinformatics* **20**, 289-290 (2004).

29 Huang, X. *et al.* A map of rice genome variation reveals the origin of cultivated rice. *Nature* **490**, 497 (2012).

30 Hu, Z. *et al.* EUPAN enables pan-genome studies of a large number of eukaryotic genomes. *Bioinformatics*, btx170 (2017).
