# Supplementary material for: Genomic variation in 3,010 diverse accessions of Asian cultivated rice
Source: Nature. 2018 Apr 25;557(7703):43–9. doi: 10.1038/s41586-018-0063-9 (PMC6784863; doi:10.1038/s41586-018-0063-9)
Supplement: Supplementary file 2 — Reporting Summary [file 41586_2018_63_MOESM2_ESM.pdf]

nature research

Corresponding author(s): Zhikang Li

☐ Initial submission ☐ Revised version ☒ Final submission

Life Sciences Reporting Summary

Nature Research wishes to improve the reproducibility of the work that we publish. This form is intended for publication with all accepted life science papers and provides structure for consistency and transparency in reporting. Every life science submission will use this form; some list items might not apply to an individual manuscript, but all fields must be completed for clarity.

For further information on the points included in this form, see Reporting Life Sciences Research. For further information on Nature Research policies, including our data availability policy, see Authors & Referees and the Editorial Policy Checklist.

Experimental design

1. Sample size

Describe how sample size was determined.

We sequenced 3,024 representative rice accessions from 89 countries all over the world, including 2,466 accessions from the International Rice Research Institute (IRRI), and 558 accessions from the Chinese Academy of Agricultural Sciences (CAAS). The 2,466 accessions contributed by IRRI represent a panel that was randomly selected from a core collection of 12,000 O. sativa accessions that was established by a semi-stratified selection scheme from more than 101,000 rice accessions. The 558 accessions contributed by CAAS included a mini-core collection of 246 accessions selected from a core collection of 932 accessions established in the same way from the 61,470 O. sativa accessions , plus 312 accessions selected based on their isozyme diversity, and used as parental lines in the international rice molecular breeding network.
The 453 accessions selected for SV and gene PAV analysis are randomly distributed and we demonstrated that they can represent the population structure.

2. Data exclusions

Describe any data exclusions.

14 accessions were excluded due to either extremely low sequencing depth or large proportion of contaminants. We described each accession in detail in the Supplementary Notes.

3. Replication

Describe whether the experimental findings were reliably reproduced.

Replication of this study's findings was not attempted.

4. Randomization

Describe how samples/organisms/participants were allocated into experimental groups.

The 453 accessions with sequencing depth >20x and mapping depth >15x were selected for SV and gene PAV analysis are purely based on sequencing depth and they are randomly distributed. We demonstrated that they can represent the population structure.

5. Blinding

Describe whether the investigators were blinded to group allocation during data collection and/or analysis.

No blinding needed. All rice sequencing data are processed equally.

Note: all studies involving animals and/or human research participants must disclose whether blinding and randomization were used.

6. Statistical parameters

For all figures and tables that use statistical methods, confirm that the following items are present in relevant figure legends (or in the Methods section if additional space is needed).

n/a	Confirmed	
☐	☒	The exact sample size (*n*) for each experimental group/condition, given as a discrete number and unit of measurement (animals, litters, cultures, etc.)
☐	☒	A description of how samples were collected, noting whether measurements were taken from distinct samples or whether the same sample was measured repeatedly
☐	☒	A statement indicating how many times each experiment was replicated
☐	☒	The statistical test(s) used and whether they are one- or two-sided (note: only common tests should be described solely by name; more complex techniques should be described in the Methods section)
☐	☒	A description of any assumptions or corrections, such as an adjustment for multiple comparisons
☐	☒	The test results (e.g. *P* values) given as exact values whenever possible and with confidence intervals noted
☐	☒	A clear description of statistics including central tendency (e.g. median, mean) and variation (e.g. standard deviation, interquartile range)
☐	☒	Clearly defined error bars

See the web collection on statistics for biologists for further resources and guidance.

Software

Policy information about availability of computer code

7. Software

Describe the software used to analyze the data in this study.	FastQC v0.11.2; BWA v0.7.10; samtools v1.0; Picatd tools release 1.119; GATK release 3.2-2; SnpEff; MUMmer v3; EMMAX algorithm implemented in SNP & Variation Suite v8.4.0; R 'qqman' package v.0.1.3; PLINK v1.90b1g; PHYLIP v3.695; 'cmdscale' function in R v.3.3.1; ADMIXTURE v.1.3; CLUMPP v.1.1.2; R v.3.3.1, with custom scripts at https://github.com/dchebotarov/3k-SNP-paper; tailed novoBreak, avaliable at https://sourceforge.net/projects/novobreak/?source=navbar ; SOAPdenovo2; GapCloser v1.1.2; Qualimap v2.026; BUSCO,with Augustus 3.2.3, hmmer 3.1b; FALCON; Canu; Quiver; Pilon; Genome Puzzle Master; bamUtil v1.0.12; CD-HIT v4.6; BEDtools v2.17.0; MAKER 2, with SNAP, AUGUSTUS; HISAT2 version 2.0.1-beta; OrthoMCL v2.0; kmer_count; BLAST v2.2.28+; mega-BLAST We described the usage of each software together with the command line in detail in the Supplementary Notes.

For manuscripts utilizing custom algorithms or software that are central to the paper but not yet described in the published literature, software must be made available to editors and reviewers upon request. We strongly encourage code deposition in a community repository (e.g. GitHub). *Nature Methods* guidance for providing algorithms and software for publication provides further information on this topic.

Materials and reagents

Policy information about availability of materials

8. Materials availability

Indicate whether there are restrictions on availability of unique materials or if these materials are only available for distribution by a for-profit company.	No unique materials were used.

9. Antibodies

Describe the antibodies used and how they were validated for use in the system under study (i.e. assay and species).	No antibodies were used.

10. Eukaryotic cell lines

a. State the source of each eukaryotic cell line used.	No eukaryotic cell lines were used.
b. Describe the method of cell line authentication used.	No eukaryotic cell lines were used.
c. Report whether the cell lines were tested for mycoplasma contamination.	No eukaryotic cell lines were used.
d. If any of the cell lines used are listed in the database of commonly misidentified cell lines maintained by ICLAC, provide a scientific rationale for their use.	No eukaryotic cell lines were used.

Animals and human research participants

Policy information about studies involving animals; when reporting animal research, follow the ARRIVE guidelines

11. Description of research animals

Provide details on animals and/or animal-derived materials used in the study.	No animals were used.

Policy information about studies involving human research participants

12. Description of human research participants

Describe the covariate-relevant population characteristics of the human research participants.	The study didn't involve human participants.
